# Distribution patterns of fern species richness along elevations the Tibetan Plateau in China: regional differences and effects of climate change variables

**DOI:** 10.3389/fpls.2023.1178603

**Published:** 2023-05-09

**Authors:** Muhammad Umair, Xiaofei Hu, Qi Cheng, Shahzad Ali, Jian Ni

**Affiliations:** College of Life Sciences, Zhejiang Normal University, Jinhua, China

**Keywords:** ferns, elevation a.s.l, unimodal pattern, species richnes, moisture index, mean annual precipitation (MAP)

## Abstract

Because of its distinct geological history, frigid temperature, and rich biodiversity, the Tibetan Plateau gives an excellent opportunity to assess the effect of climate change on determining species richness. The distribution patterns of fern species richness and their underlying processes have long been a matter of debate in ecology research, with various hypotheses suggested over the years. Here, we explore richness patterns of fern species in Xizang on the southern and western Tibetan Plateau along an elevational gradient (100–5300 m a.s.l.) and evaluate climatic factors causing the spatial decrease and increase of fern species richness. We used regression and correlation analyses to relate the species richness with elevation and climatic variables. Throughout our research, we identified 441 fern species from 97 genera and 30 families. The Dryopteridaceae family (S = 97) has the highest number of species. All energy-temperature and moisture variables except drought index (DI) had a significant correlation with elevation. The altitude has a unimodal relationship with fern species, and the species richness is the largest at an altitude of 2500 m. The horizontal richness pattern of fern species on the Tibetan Plateau also showed that areas of extremely high species richness are mainly distributed in Zayü and Mêdog County, with an average elevation of 2800 m and 2500 m, respectively. The richness of fern species has a log-linear relationship with moisture-related factors such as moisture index (MI), mean annual precipitation (MAP), and drought index (DI). Because the peak corresponds spatially with the MI index, the unimodal patterns confirm the significance of moisture on fern distributions. Our results showed that mid-altitudes have the highest species richness (high MI), but high elevations have lower richness due to high solar radiation, and low elevations have lower richness due to high temperatures and low precipitation. Twenty-two of the total species are classified as nearly threatened, vulnerable or critically endangered, and varied in elevation from 800 m to 4200 m. Such relationships between the distribution and richness of fern species and climates on the Tibetan Plateau can provide data support for future predictions of the impacts of climate change scenarios on fern species, the ecological protection of representative fern species, and references for the planning and construction of nature reserves in the future.

## Introduction

The distribution patterns of species richness and their underlying processes have long been studied in ecology (Rosenzweig, 1995; Gaston, 2000). Over the past years, numerous studies have examined the relationships between elevational gradients and species richness to fully understand the nature of such gradients and to develop more efficient conserving strategies for biodiversity under climate change (Lomolino, 2001; Körner, 2007). Species richness frequently exhibit complicated interactions with elevation that differ depending on the taxonomic category and levels of gradient (Rahbek, 1995). Three types of diversity patterns have been identified: a diversity plateau at low altitudes, a mid-elevation peak with an intermediate level of richness, and a steady reduction in species richness with elevation (Rahbek, 1995; Rahbek, 2005). A mid-elevation curve is the most prevalent, as shown in almost half of previous studies, and is much more common than the monotonically declining pattern (Rahbek, 1995; Rahbek, 2005). Several interrelated factors contribute to species richness trends along elevation gradients (Kessler et al., 2011). Ecologists have generally agreed that climate change has a significant role in shaping how species are distributed along the gradient (Bhattarai et al., 2004; Acharya et al., 2011; Kessler et al., 2011).

Climate change is more significant than other ecological factors in explaining the pattern of species richness along the elevation gradient in the Tibetan Plateau (TP), China (Liang et al., 2020). The most significant factor influencing the richness and distribution patterns of species along altitudinal gradients is thought to be climatic because climatic variables are significantly correlation with elevation (Vetaas and Grytnes, 2002; Wu et al., 2013). Species richness increased in areas where topography and elevation caused climatic changes (Körner, 2003). Understanding the mechanisms that generate patterns of species richness along altitude gradients is therefore critical in evaluating the effects of climate change on biodiversity. Community ecologists have proposed several hypotheses to investigate the species richness pattern and the reasons that regulate it, taking altitudinal gradient and climatic variables into account (Pandey et al., 2020). Precipitation, temperature, humidity, potential evapotranspiration, growth season duration, and solar radiation are all climatic factors that have been hypothesized as major contributors in the distribution of plants on mountains (Funnell and Parish, 2001; Baniya et al., 2010; Acebey et al., 2017). Thus, in this work, we looked at the impact of energy and water, in understanding richness patterns of fern species on TP. The “energy-water hypothesis” is the most prevalent and widely debated explanation for explaining fern species richness trends. Fern species have previously discussed the link between species richness, water, and energy (Hawkins et al., 2003; Bhattarai et al., 2004; O'Brien, 2006). This concept is based on the ability of plants to acquire water and energy. The hypothesis related to energy-water dynamics is extremely important in understanding the patterns of fern species richness along elevation gradients.

Climate change is likely to have a significant impact on fern species (de Gasper et al., 2021), particularly epiphytic ferns (Hsu et al., 2012; Hsu et al., 2014). This is not unexpected because epiphytes are thought to be more susceptible to fluctuating and harsh climatic conditions at different phases of their development (Benzing, 1998; Klinghardt and Zotz, 2021). Several fern species that have evolved to specific climatic niches may face new problems as a result of aversive effects of climate change (Anderson, 2018). When climatic variables surpass a species’ physiological tolerances, they directly or indirectly influence the distribution range limits of certain species (Bachman et al., 2004; Rowe, 2009). Recent research has discovered that the richness of fern species along elevational gradients is typically connected with climates such as temperature and precipitation (Bhattarai et al., 2004; Tang et al., 2014). Climatic variables that change with elevations and can all have a significant impact on the distribution of fern species throughout the elevational gradient (Körner and Kèorner, 1999). Individual species’ distribution margins in the elevational gradient are defined indirectly or directly by excessive extremes of these climate variables. Many variables, such as temperature, precipitation, solar radiation, alter with elevation in mountainous areas, providing unique opportunities to study how biological diversity responds to such climate variables within geographically constrained areas (Körner and Kèorner, 1999; Körner, 2007).

Ferns have a total of 11,500 species around the world and are most diverse in tropical and subtropical mountains (Smith et al., 2006). Climate change and habitat destruction may result in a significant loss of fern biodiversity (Anderson, 2021). Ferns provide important ecological services and are economically useful in the horticulture sector. Ferns use microscopic spores to disperse and establish new populations in isolated places (Rose and Dassler, 2017), and they are highly sensitive to air humidity and temperature (Pouteau et al., 2016). Moreover, ferns are typically more sensitive to a limited water supply (Hernández-Rojas et al., 2020) because their stomatal control is less effective than that of angiosperms (Brodribb and McAdam, 2011), making them useful climatic indicators. Ferns have produced some of the most dramatic mid-elevation peaks of species richness at mid-altitudes (Hemp, 2001; Kessler, 2001; Bhattarai et al., 2004; Kluge et al., 2006; Watkins et al., 2006; Kessler et al., 2011; Marini et al., 2011). High air humidity and mild temperatures at mid-altitudes are typically linked with maximum fern richness (Hemp, 2001; Hemp, 2002; Bhattarai et al., 2004; Kluge et al., 2006). Despite their extensive diversification and specific adaption, ferns remain to be a particularly valuable class of vascular plants for scientific study. Due to widespread habitat degradation, anthropogenic climate change, and human exploitation, many fern species are in risk of extinction (such as agricultural extension, timber harvesting and urban development in natural environments) (Arcand and Ranker, 2013; Nowicki and Kowalska, 2018). However, research on the distribution patterns of fern species richness along the elevational gradients on the Tibetan Plateau (the highest plateau in the world), regional differences, and their drivers is necessary to improve our understanding of these issues and provide insight into biological conservation.

The fern species richness is evaluated in order to generate hypotheses about the numerous climatic variables that cause changes in species richness over the entire elevational gradient, as well as to study possibly distinct influences of the same variable at lower and higher elevations. Many ecological and evolutionary factors play a significant role in the assembly of ferns into local communities from a regional pool of species (Qian et al., 2023). While many variables impact the regional species pools from which fern species evolve, including climate and, to a lesser extent, geomorphology and soils. These factors operate as environmental filters, selecting which species are able to withstand the pressures posed by climate change and which are best equipped to capitalize on the chances for dispersion, development, and reproduction (Coyle et al., 2014; Qian et al., 2023). However, the main objective of this research is to (1): investigate regional variations in richness patterns of fern species and their conservation status along altitudes (2), evaluate the predictive capacity of energy-water relation in explaining the distribution pattern of fern species richness along altitudinal gradients, and (3) demonstrate how climatic variables may determine variation in fern richness along the entire gradient ranged from 100−5300 m, as well as along two sub-gradients, namely, low sub-gradients (LSG)=100−2500 m and upper sub-gradients (USG)=2500−5300 m on TP.

## Material and methods

### Study area and species data

The Tibet Plateau covers an area of about 2.3 million square kilometers, is located between Central Asia and Southeast Asia, and has an average altitude of over 4,000 meters (**Figure S1**). It is the highest and largest plateau on Earth and is known as the roof of the world because it is surrounded by some of the highest mountains on Earth, including the Pamirs, Kunlun Mountains, and Himalayas. Many studies provide convincing evidence that the Tibetan Plateau has significant dynamic and thermal influences on regional temperature and weather patterns in the Northern Hemisphere as well as on air circulation. The annual average temperature is -15°C~10°C, and the annual average precipitation is 392~764 mm. Snowfall in winter is infrequent, and snow melts quickly, at least on sunny slopes (Miehe et al., 2008). After the Antarctic and the Arctic, the “Third Pole” is the region with the highest diversity of biological species, ecological types and climate types in the world (Fu et al., 2010; Gao et al., 2016). The main types of vegetation on TP are alpine shrub, alpine steppe (mainly cushion plants), and alpine meadow (mostly perennial grasses) (Liu et al., 2019; Liu et al., 2021). The TP is particularly susceptible to climate change and environmentally fragile (Pan et al., 2022). As a biodiversity hotspot (Tang et al., 2006; López-Pujol et al., 2011), TP provides habitat for more than 9000 species of vascular plants, of which more than 18% are indigenous to the region (Wu, 2008); the diverse habitats also show that species richness varies greatly over the plateau (Tang et al., 2006; Yang et al., 2013).

In this study, the Xizang Autonomous Region is selected as the study area, because the fern species are mostly distributed in Xizang in the southern and western TP. We used the elevational range data of fern species from “The vascular plant and their ecogeographical distribution of the Qinghai-Tibetan Plateau” (Wu, 2008). These inventories and checklists are based on plant and distribution data on TP collected over the period of the last 60 years by large numbers of national and local teams (Sun, 2007; Zhang, 2013). In order to make the database more accurate, we also verified the species record (https://www.plantplus.cn/), nature reserve checklists, scientific papers, relevant provincial records, and various local floras in the area. According to a recent review of specimen collection integrity, practically all counties on the TP have been thoroughly studied (Yang et al., 2013). According to our preliminary research, all fern species on the TP were dispersed within an elevation range of 100 m and 5300 m. The elevation gradient of fern species distribution was divided into 53 elevation bands between 100 m and 5300 m (each 100 m). Every 100 m between a species’ upper and lower levels of elevational gradients was designated as its presence. For example, a species with an elevation limit of 1850 to 2270 m can be found in the elevation bands 1900, 2000, 2100, 2200, and 2300 m [8, 40]. This yields an estimated gamma diversity, which is the total richness of an altitudinal zone (Lomolino, 2001) (originally introduced by Grytnes and Vetaas (2002)). Because fern species diversity is frequently employed for indexes integrating evenness and richness, however, we define fern richness or species richness as the number of fern species present in each 100-m unit.

### Species richness

The response variable in this investigation is species richness, which is defined as the number of fern species present in each elevation band as determined by interpolation methods (McCain, 2004). The existence of species was approximated based on the elevational range of the species from its maximum and minimum elevation distribution. This strategy presumed that taxa may be found at any height between their lowest and highest peak. The interpolation approach is also useful in resolving the under-sampling problem. Furthermore, the surface area of the mountain will be greater at the bottom than at the peak due to the hump-shaped design (Körner, 2003). The surface area of each band will differ from one another, and species richness fluctuates as land area changes across elevation (Rosenzweig, 1995). The area inside each elevation band is also a proxy variable for the size of the gene pool and can have a direct impact on species richness (Hu et al., 2018). The elevation and area data were downloaded from the Shuttle Radar Topography Mission (SRTM; https://www2.jpl.nasa.gov/srtm/).

Each species’ geographic range is described in two domains: elevation range (the lower and upper elevation) and horizontal distribution at the county level. In Xizang, there are 139 counties with a wide range of terrain. To reduce the potential influence of area on the evaluation of species richness, the distribution maps of each species were subsequently converted to grid-based maps. To do so, we divided the plateau into 1,139,621 1 km × 1 km girds. We then combined this grid map with a DEM (at 1 arc−2^nd^ resolution) and a Chinese administrative map to establish the particular grid point and elevation for each grid. A species was identified as being present in the grid when both its horizontal and vertical distribution were taken into account. Because some grids included multiple counties, converting only on horizontal distribution would have enlarged the species’ range of distribution. This bias, however, has been diminished for the two reasons indicated below: First, there is a difference in size between a grid than a county; second, we provided an elevation range for each species. The term “species richness” refers to the total number of species in each grid.

### Climatic variables

The elevation directly or indirectly determines the gradient in environmental variables, while these environmental gradients have a direct relationship with the growth and development of plants (Kluge et al., 2006) Long-term climate records from 1981 to 2010 at 2152 meteorological stations across China were averaged to produce climate data for five variables (monthly precipitation, temperature, sunshine percentage, and the absolute maximum and minimum temperatures, **Figure S2**) (http://data.cma.cn). Based on the elevation data model of the “SRTM” (Shuttle Radar Topography Mission), these five climate variables were projected into 1 km grids using a surface fitting method of thin plate smoothing spline (ANUSPLIN version 4.4, Xu and Hutchinson (2013)), which brought took the impact of elevation on climates into account (Farr et al., 2007). We used eight climatic variables to evaluate the pattern of fern species richness along elevation gradients. The annual growing degree days above 0°C (GDD_0_) and 5°C (GDD_5_), mean annual temperature (MAT), mean annual precipitation (MAP), growing season precipitation (GP), sunshine percentage (SSP%), annual drought index (DI = 1-AET/PET), and annual moisture index (MI = MAP/PET), were all calculated using the interpolated climate data (Gallego-Sala et al., 2010). AET stands for annual actual evapotranspiration and PET stands for annual potential evapotranspiration measured using the Penman-Monteith technique.

### Statistical analysis and correlation

Primarily, the elevational gradient was utilized as an explanatory variable, while species richness was used as a response variable. We used Pearson correlation analysis to see whether there was a significant association between elevation, species richness, and climate factors. Analyzing the richness pattern at the gradient’s two ends may provide further light on the major variables influencing species interactions. The total gradient was separated into sub-levels of 100-2500 m (lower sub-gradient, LSG) and 2500-5300 m (upper sub-gradient, USG), and assessed both independently and as a whole, based on the mid-elevation peak at 2500 m. To correlate the fern richness with climatic factors and elevation, we employed a generalized linear model (GLM). The popular GLM method is used to correlate climatic factors to species richness. A logarithmic connection is necessary because species richness is discrete (counting) variable with a Poisson distribution error. An identity link function was used to test the regression models as well (which assumes a normal distribution of errors). Each variable was evaluated independently. Up to 3^rd^ order polynomials were used to test the models. These polynomials were evaluated both individually and in relation to null models. By analyzing the normalized residuals to the fitted values and utilizing normal probability plots, it was possible to determine whether the fitted statistics were appropriate.

We performed principal component analysis (PCA) to assess the difference between two sub-gradients (LSG and USG) to determine the significant association between climatic factors and fern richness. The altitudinal gradient was employed as an independent variable, whereas climatic parameters and fern richness were used as dependent variables. The association between fern species richness and climate factors with altitude was shown using Heatmap (Lomolino, 2001) in R software 3.6.3. Microsoft Excel 2010 (Microsoft, Redmond, WA, USA), R software 3.6.3, and PAST 3.20 (Hammer et al., 2001) were used for all graphical data analysis.

## Results

### Regional variations in the richness patterns of fern species and their conservation status

**Figure 1:**
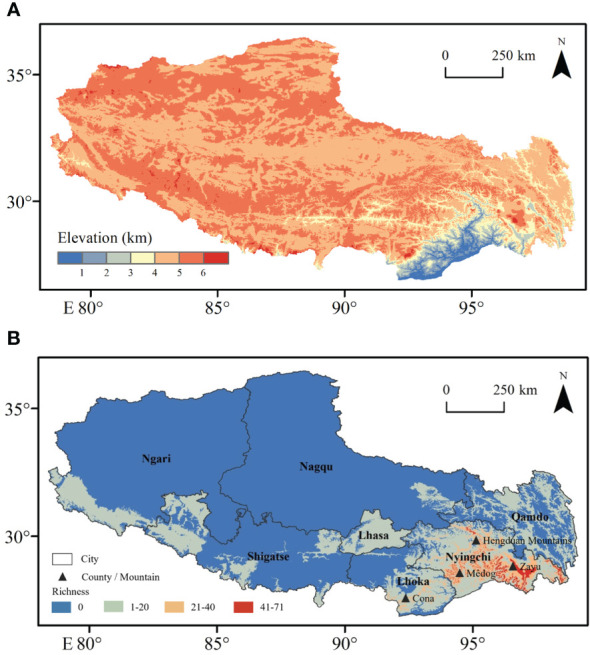
Distributions of **(A)** digital elevation model and **(B)** fern species richness in Xizang, southern and western Tibetan Plateau.

**Figure 2:**
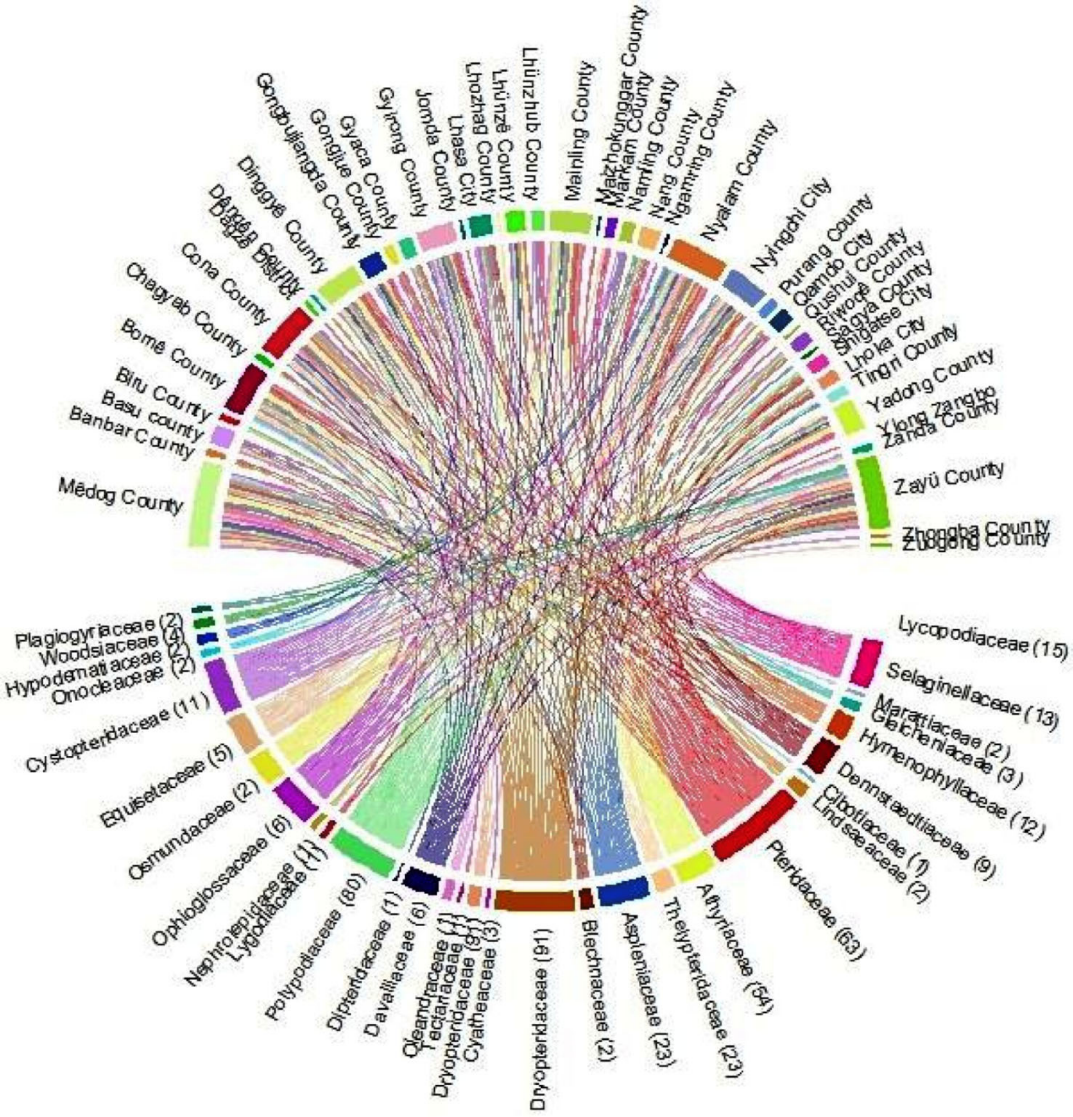
Regional distribution of the families of fern species in Xizang, Tibetan Plateau, China.

**Figure 3:**
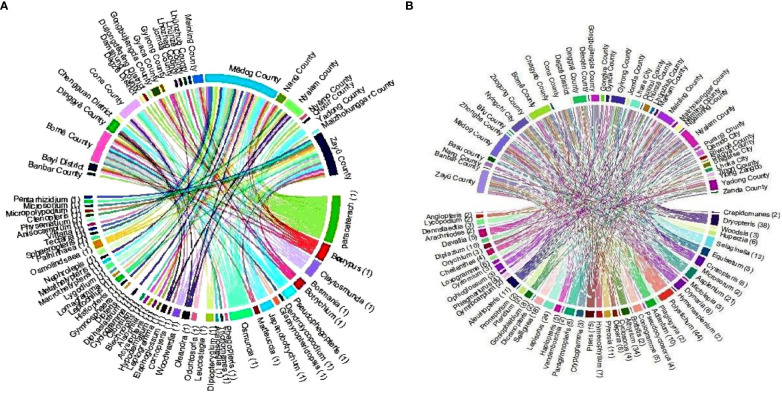
Regional distribution of the **(A)** genera (S = 1) and **(B)** genera (S > 1) of fern species in Xizang, Tibetan Plateau, China.

Under climatic conditions, the area of fern species with extremely high richness accounts for 99.9% of the area of Xizang (**Figure 1**), which is mainly distributed in Nyingchi city (Zayü County, Mêdog County, Bomê County, and Bayi County). The Zayü County with high fern richness accounts for 70.14% of the area of the Nyingchi city, followed by Mêdog County (26.73%), Bomê County (3.02%), and Bayi County (0.02%). Similarly, the area with high richness accounts for 0.09% of the area of Xizang, mainly distributed in Qamdo city (**Figure 1**). On the other hand, the low richness areas are Shigatse, Lhoka, Lhasa, Ngari, and Nagqu cities (**Figure 1**). The ferns are distributed between 100 m and 5300 m in Xizang region of the TP. There are 441 species overall, 30 families and 97 genera (**Figures 2**, **3**). The most species-rich family, i.e., Dryopteridaceae (S = 91), is mainly distributed in Nyingchi city, Qmado city, Shigatse city, Lhasa city, Lhoka city, Nagqu city (Biru County), and Ngari city (Purang County) (**Figure 2**). There are only 32 species above 4000-5300 m, representing 15 genera and 9 families (**Figure S3**). The most species-rich genus is *Polystichum* (S = 44), which is mainly distributed in Nyingchi city, Qmado city, Shigatse city, Lhasa city, and Lhoka city (**Figure 3A**). While 48 genera containing only single species (**Figure 3B**).

It was noted that 53% of fern species are least concerned (LC), 29.2% of species are listed as data deficient (DD), 12.7% of species are not evaluated (NE), 3.2% of species are listed as near threatened (NT), 0.91% of species (i.e., *Huperzia selago, Anisocampium cuspidatum, Dryopteris lachoongensis*, and *Drynaria delavayi*) are vulnerable (V), and 0.91% of species (i.e., *Huperzia serrata, Gymnosphaera andersonii, Sphaeropteris brunoniana*, and *Selliguea dareiformis*) are listed as endangered (EN) by IUCN (International Union of Conservation of Nature) (**Figure S4**).

### Fern-climate relationships along the elevational gradients

**Figure 4:**
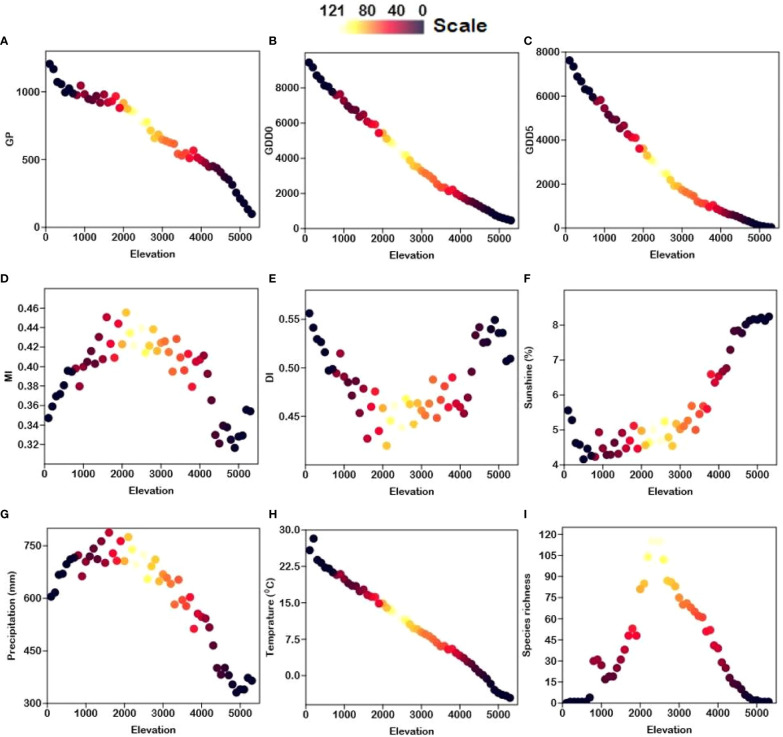
Scatter plots showing the relationship between elevation (m) and climatic variables i.e., **(A)** GDD_0_ (growing degree days of daily temperature >0°C), **(B)** GDD_5_ (growing degree days of daily temperature >5°C), **(C)** MAT (mean annual temperature), **(D)** MAP (mean annual precipitation), **(E)** GP (growing degree days), **(F)** SSP (sunshine %), **(G)** MI (moisture index), **(H)** DI (drought index), and **(I)** S (species richness). The scale indicates fern species richness in Xizang, Tibetan Plateau, China.

**Figure 5:**
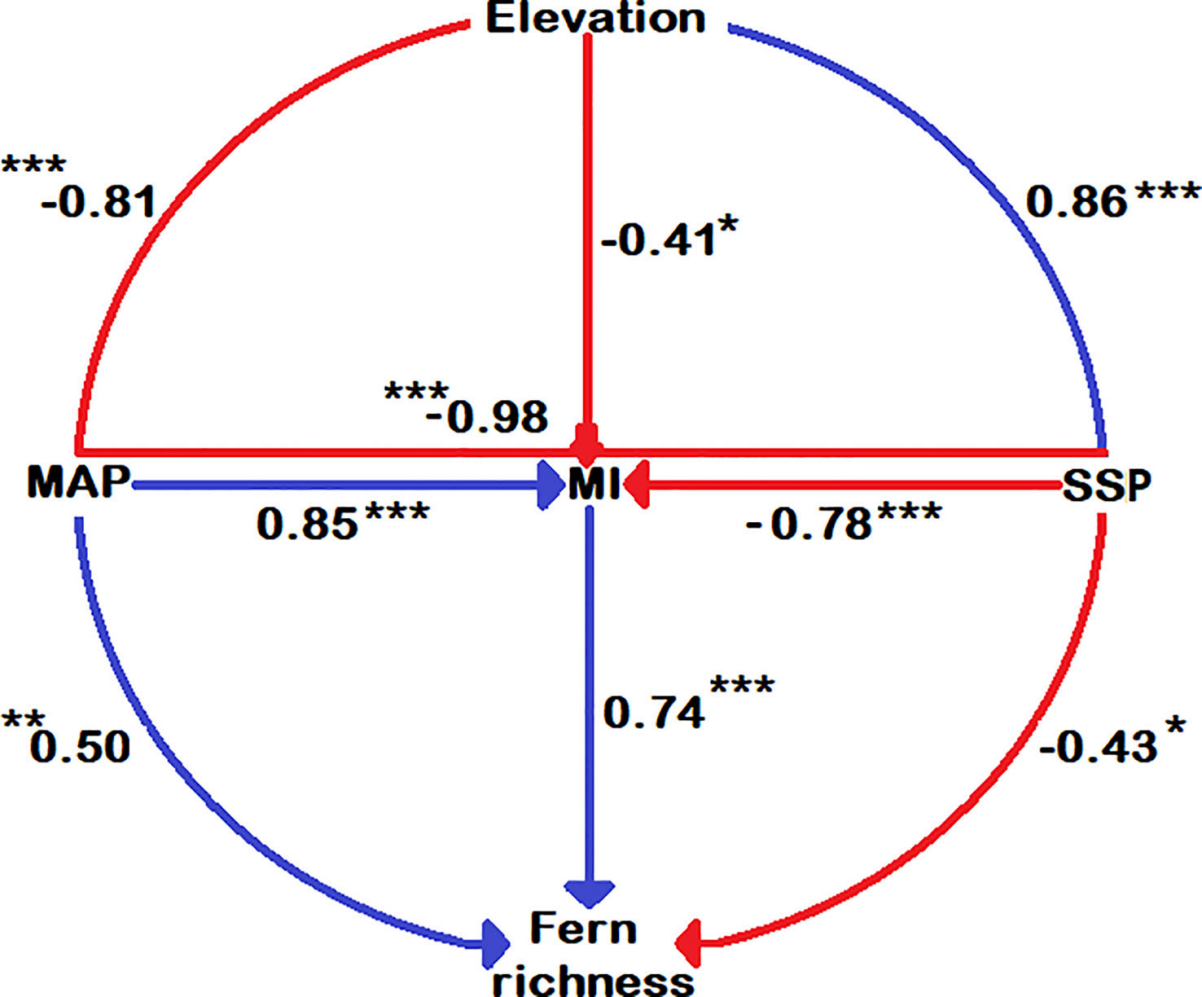
Correlation among climatic variables, species richness and elevations. The significance level is shown by the star symbol; * significant at 0.05, ** significant at 0.01, and *** significant at 0.001.

Except for DI, all energy-temperature and moisture variables have a strong correlation with elevation (**Supplementary Table 2** and **Figure 4**). The highest fern richness is found at around 2500 m. (**Figure 4**). **Figure 5** shows that overall fern species richness has a significant positive correlation with MI (r = 0.74) and MAP (r = 0.50) and a significant negative correlation with DI (r = -0.77) and SSP (r = -0.43). Total fern species richness has no significant correlation to other climate gradients.

**Figure 6:**
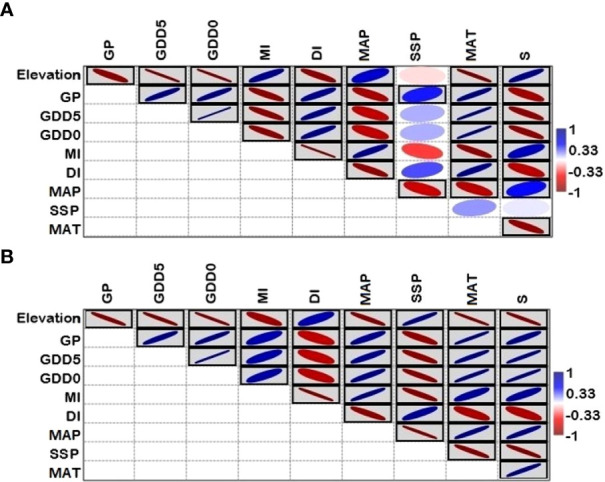
Pearson correlation among climatic variables, species richness and elevation: **(A)** lower elevation sub-gradient (100-2500 m a.s.l.), **(B)** upper sub-gradient (2500-5300 m a.s.l) in Xizang, Tibetan Plateau, China.

**Figure 7:**
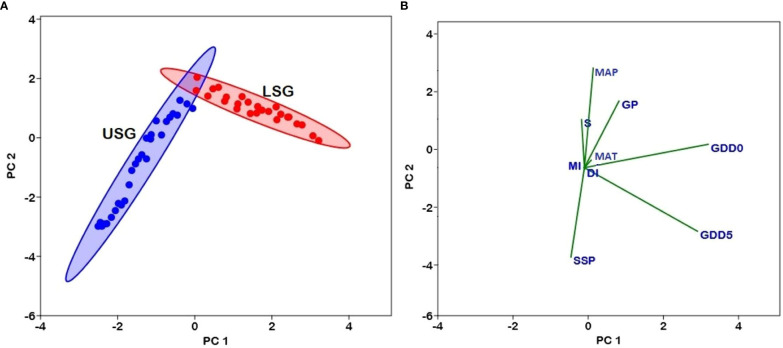
**(A)** Plot of variables in the PCA conducted with GDD5 (growing degree days of daily temperature >5°C), GDD0 (growing degree days of daily temperature >0°C), MAT (mean annual temperature), GP (growing degree days), MAP (mean annual precipitation), SSP (sunshine %), MI (moisture index), DI (drought index), and S (species richness) in lower sub-gradients (LSG) and upper sub-gradients (USG) in Xizang, Tibetan Plateau. The subgradients LSG and USG are indicated by different colors (Red circles–LSG and Blue circles–USG). **(B)** Loadings of variables in PCA show the correlation with PC1 and PC2.

**Figure 8:**
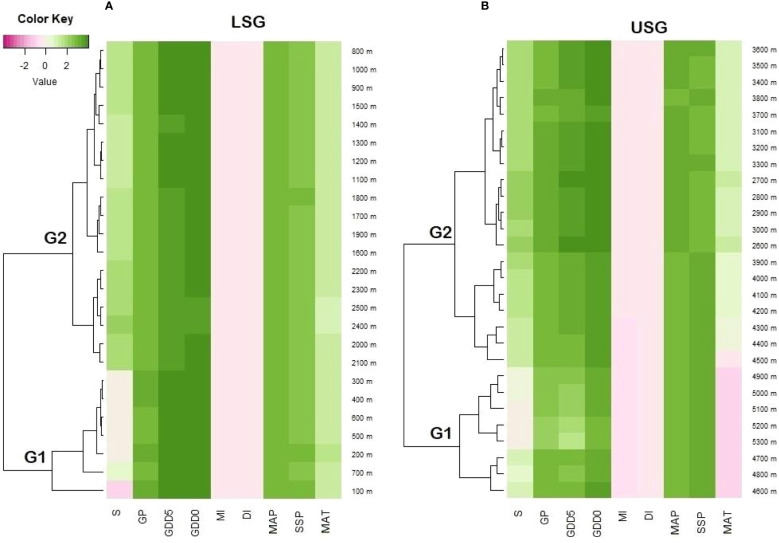
Heatmap shows the relationship between elevation and different climatic variables i.e., GDD5 (growing degree days of daily temperature >5°C), GDD0 (growing degree days of daily temperature >0°C), MAT (mean annual temperature), GP (growing degree days), MAP (mean annual precipitation), SSP (sunshine %), MI (moisture index), DI (drought index), and S (species richness) **(A)** lower elevation sub-gradient 100-2500 m a.s.l. **(B)** upper subgradient 2500-5300 m a.s.l. on the Tibetan Plateau, China. G1 and G2 represent the elevation groups based on cluster analysis.

The associations between elevation and fern richness exhibit substantial positive (r = 0.92) and negative (r = -0.99) correlations when separated into two gradients of 100-2500 and 2500-5300 m, respectively (**Figure 6**). All energy-temperature variables and moisture variables had significant correlation with elevation and fern richness (**Figure 6**). According to Principal component analysis (PCA), the first two axes have a variance of 99.3%, while the “PC1-axis” and “PC 2-axis” have variations of 94.6% and 4.78%, respectively (**Figure 7A**). **Figure 5B** demonstrates the positive correlation between the PC 1-axis and the variables GDD_5_ (r = 0.66), GDD_0_ (r = 0.72), and DI (r = 0.0007). GP (r = 0.46), MAT (r = 0.01), MAP (r = 0.66), MI (r = 0.05), and S (r = 0.33), on the other hand, are positively correlated with the PC 2-axis, and SSP (r = -0.05) is negatively correlated with the PC 2-axis (**Figure 7B**). Based on climatic variables and fern richness, cluster analysis revealed two groups of different elevation gradients in USG and LSG on the basis of similarity (**Figure 8**). In LSG, the first cluster G1 (100 m - 700 m) had lower fern species richness, whereas the second cluster G2 (700 m - 2500 m) contained higher number of fern species. On the other hand, in USG, the first cluster G1 (2500 m - 4600 m) had higher fern species richness, whereas the second cluster G2 (4600 m - 5300 m) contained lower number of fern species.

**Table 1** depicts the correlations between fern species richness and climate factors. In general, species richness is unimodally related to temperature-related variables such as MAT, GDD_0_, and GDD_5_ (**Table 1**). Because these temperature-related variables MAT (r = -1.00), GDD_0_ (r = -0.99), GDD_5_ (r = -0.98), and GP (r = -0.98) are highly correlated, the fern richness trend along MAT, GDD_0_, GDD_5_, and GP is the same as the elevation trend (**Table 1** and **Figure 4**). Fern species richness declines monotonically with increasing and decreasing values of MAT, GDD_0_, GDD_5_, and GP. There is a log-linear trend between the moisture-related variables (i.e., MAP, MI, and DI) and fern richness. Because fern richness along elevation gradients is significantly correlated with both the MI (r = 0.74) and DI (r = -0.74) variables (**Table 1** and **Figure 4**). For fern species, the highest MI values are between 0.43 and 0.45 (**Figure 4**), which appears around 2500 m. The fern species richness along the MAP gradient displays a log-linear trend. Along the two sub-levels (LSG and USG) also shows a log-linear relationship with MAP (**Figure S5** and **Table 1**). There is a log-linear association between SSP and fern richness over the whole gradient, however above 2000 m, there is a negative log-linear trend. There is no substantial pattern below 2000 m (**Figure S5** and **Table 1**).

**Table 1:** Summary of regression statistics for the species richness relationship between variables along the whole elevation gradient and two sub-gradients.

Predictors	Range	GLM order	d.f.	R^2^%	P (F)
**GP**	100-5300	2	26.235	51.21%	P < 0.001
	100-2500	1	51.51	69.13%	P < 0.001
	2500-5300	1	175.23	87.08%	P < 0.001
**GDD_5_**	100-5300	2	39.357	61.15%	P < 0.001
	100-2500	1	109.53	82.65%	P < 0.001
	2500-5300	1	644.25	96.12%	P < 0.001
**GDD_0_**	100-5300	2	62.643	71.48%	P < 0.001
	100-2500	1	112.09	82.98%	P < 0.001
	2500-5300	1	809.08	96.89%	P < 0.001
**MI**	100-5300	1	60.445	54.24%	P < 0.001
	100-2500	1	27.056	54.05%	P < 0.001
	2500-5300	1	85.552	76.69%	P < 0.001
**DI**	100-5300	1	73.197	58.94%	P < 0.001
	100-2500	1	29.808	56.45%	P < 0.001
	2500-5300	1	47.673	64.71%	P < 0.001
**MAP**	100-5300	1	16.757	24.73%	P < 0.001
	100-2500	1	6.849	22.95%	P < 0.001
	2500-5300	1	283.16	91.59%	P < 0.001
**SSP**	100-5300	1	11.461	18.35%	P = 0.001
	100-2500	1	0.058351	0.25%	P = 0.811
	2500-5300	1	378.45	94.39%	P < 0.001
**MAT**	100-5300	2	46.251	64.91%	P < 0.001
	100-2500	1	85.372	78.78%	P < 0.001
	2500-5300	1	437.5	94.39%	P < 0.001

The numbers 1, 2, and 3 indicate first, second, and third order polynomials. Climatic variables that are used to evaluate the pattern of fern species richness along elevation gradients were GDD_5_ (growing degree days of daily temperature >5°C), GDD_0_ (growing degree days of daily temperature >0°C), MAT (mean annual temperature), GP (growing degree days), MAP (mean annual precipitation), SSP (sunshine %), MI (moisture index), DI (drought index).

## Discussion

### Richness pattern of fern species along the elevational gradient

Fern richness was higher in the mountains and valleys along the plateau’s south and east sides, where elevation was low, than in the basins along the plateau’s northern border and the high-latitude (above 4000 m) region (**Figure 1**). According to Zheng, Zhang and Wu (Zheng et al., 2000), the southern slopes of Tibet and the south Hengduan Mountains are both tropical and subtropical, accounting for approximately 61.2% of the total fern genera on the plateau.

Several studies have noticed the pattern of fern species richness along altitudinal gradients and their underlying processes in various regions in recent years. The species richness showed variation with elevation in TP, China (Wang et al., 2006; Wang et al., 2007). Bhattarai, Vetaas and Grytnes (Bhattarai et al., 2004) also reported the fern species richness varies strongly with elevation in Himalayan region of Nepal. According to some studies, the highest richness of species appears at the mid-elevation peak (Bhattarai et al., 2004; Wang et al., 2007), whereas other studies show that species abundance increases or decreases continuously as elevation increases (Sanders, 2002; Shimono et al., 2010; Subedi et al., 2020). Nonetheless, there are two types of diversity patterns that are commonly observed (1): a steady reduction in species richness with elevation [46], and (2) a mid-elevation peak with an intermediate level of richness [25]. However, there are two most common forms of diversity patterns (Gaston, 2000); monotonically decreasing curve with increasing altitude (Wang et al., 2006), and (Rosenzweig, 1995) a mid-elevation peak with high diversity at intermediate altitudes (Marini et al., 2011). In our study, fern species showed high richness at mid-elevation peak. Fern richness reaches a maximum at 2500 m and decreases constantly as elevation gradients increase and decrease. Some regions with high fern species richness at mid-elevation include the Himalayan region, Nepal (Bhattarai et al., 2004), Tahiti, France (Pouteau et al., 2016), NE Italy (Marini et al., 2011), and Costa Rica (Watkins et al., 2006).

The mechanisms defining a species’ mid-elevation peak are unknown, although there are four major elements involved in the decline of fern species richness i.e., moisture availability (air humidity), rainfall, the solar radiation; and hard boundaries, such as the lack of tree-habitats above timberline. Hard boundaries are imposed in terms of species distribution level and survival resistance (Colwell and Hurtt, 1994). If these limits are resistant to dispersal of species, they are referred to as hard boundaries. According to some researchers, species richness may peak at mid-elevation due to hard boundaries that exist at both extremes of the elevational gradient (Colwell and Hurtt, 1994; Vetaas and Grytnes, 2002). The mid-domain effect is often larger for broadly dispersed species (species with extensive ranges) than for narrowly distributed species (with small ranges) (Colwell et al., 2004). The abrupt decline in species richness over 4000 m may be explained by a dynamic hard barrier regulated by climate (Vetaas and Grytnes, 2002). According to Bhattarai, Vetaas and Grytnes (Bhattarai et al., 2004), climatic variables, not hard boundaries, are the primary reasons of the unimodal pattern.

Ferns reach their highest elevation at 5300 m on the TP. According to Liang, Wang, Piao, Lu, Camarero, Zhu, Zhu, Ellison, Ciais and Peñuelas (Liang et al., 2016), TP has the highest alpine treelines in the Northern Hemisphere (4900 m a.s.l). Our findings revealed the presence of three species in the alpine environment, including *Cystopteris dickieana, C. fragilis*, and *Polystichum lachenense*, suggesting that alpine ferns are mostly sink communities of alpine species that can scarcely endure the harsh and stressful open habitat (Grytnes, 2003). The mechanism of inadequate control of evaporative potential of fern species in harsh open alpine habitat may be explained by source-sink dynamics. Because the significant relation between tree species and fern species can help to regulate of evapotranspiration potential to counterbalance high levels of water loss. According to Grytnes (2003), If the ecological conditions are equivalent, sink populations are often formed from source populations within the territory.

Mid-elevation habitats received greater diaspore input than sites at the extremes of the elevational gradient, resulting in greater plant species richness (Bhattarai et al., 2004). Furthermore, trees defend ferns against desiccation and provide suitable environmental condition, which explains the significant correlation between fern richness and tree species at mid-elevation. As samples obtained throughout a sub-tropical elevational gradient indicated, fern species clearly declined toward the lowlands due to habitat degradation (Bhattarai and Vetaas, 2003). Stochastic mechanisms like as the mid-domain effect, which predicts that species ranges evenly distributed within a restricted region would overlap more in the middle than at the extremes of the elevational gradient (Grytnes and McCain, 2007), may influence fern species richness patterns, albeit to a lower amount than climate change (Bhattarai et al., 2004; Kluge et al., 2006; Kessler et al., 2011). Bhattarai, Vetaas and Grytnes (Bhattarai et al., 2004) observed that the reduced fern abundance may be attributed to climatic factors rather than habitat degradation.

### Pattern of fern richness and climates

The distribution of fern species richness along the elevational gradients has been shown to be strongly linked to climatic conditions at both the locals (Bhattarai et al., 2004; Kluge et al., 2006; Pouteau et al., 2016) and global scales (Kessler et al., 2011). Fern species richness is lower in arid lowlands and on cold peaks than in mid-elevation places (Bhattarai et al., 2004), and diverse climatic parameters (moisture, temperature, precipitation, and solar radiation respectively) have been proposed to restrict fern richness at both extremes of the elevational gradient (Kessler et al., 2011; Pouteau et al., 2016). Climate variables are the key drivers of the mid-elevation peak of plant species on the TP in China (Liang et al., 2020; Sun et al., 2020), which may be related to exceptional elevational range of the TP and a more comprehensive and evident vertical climatic gradient than other mountains of comparable height. The current climatic conditions appear to be having two significant effects: low temperatures and precipitation on cold summits (high altitudes) (Marini et al., 2011) and low moisture availability (humidity and precipitation) in dry regions (low altitudes) (Kessler et al., 2011). Temperature, humidity, precipitation, and solar radiation may all be used to calculate the overall productivity of an elevation gradient (McCain and Grytnes, 2010).

### Strong relation of fern richness and moisture variables

MI are highly correlated (r = 0.74) with fern richness along the elevation gradients (**Figure 5**). Since moisture is required for free-swimming motile antherozoids to proliferate, fern richness and MI showed a strongly positive log-linear relationship (Page, 2002). Another element contributing to the high moisture content at mid-elevation is the cloud zone, where massive volumes of water are rapidly deposited onto plants *via* light mist (horizontal rainfall in forest zones) and clouds (McCain and Grytnes, 2010; Hamilton et al., 1995). Moreover, it causes decreased evapotranspiration and sunshine (Hamilton et al., 1995), as well as increased atmospheric humidity (Kluge et al., 2006), which may increase fern richness (Bhattarai et al., 2004; Pouteau et al., 2016). According to Pouteau, Meyer, Blanchard, Nitta, Terorotua and Taputuarai (Pouteau et al., 2016) and Navarrete (2001) assert that rain forests at intermediate altitude have the highest fern richness because these forests have lower solar radiation, vapor pressure, as well as an overall decrease in evaporation potential. According to our research, the highest fern richness was found at the same height of 2500 m (**Figure 4I**), which also supports the highest water availability at mid-elevation areas. The horizontal richness pattern of fern species on TP also showed that areas of extremely high species richness are mainly distributed in Mêdog and Zayü County (**Figure 1**), with an elevation of 2500 m and 2800 m, respectively. Both counties has a favorable climate caused by the South Asian monsoon, which brings moisture from the Indian Ocean (Du et al., 1990; Buzhuo et al., 2000). The areas of relatively high species richness were distributed in the Lhasa City with an altitude of 3650 m and the southern section of Hengduan Mountains.

Water and energy interaction is essential for biological activity and species diversity (O'Brien, 1998; Whittaker et al., 2001; Acharya et al., 2011). Fern species richness showed a similar tend with elevation gradients, reaches a maximum at 2500 m, and decreases constantly as elevation gradients increase or decrease. This is most likely due to high MI at mid-elevations (**Figure 4G**), where there is a lot of water stored directly on plants due to higher precipitation. Ecologically, it makes sense that the highest fern abundance at 2500 m corresponds to the highest moisture availability. Moisture observations at four distinct altitudes (40, 650, 1800, and 2800 m) collected over a year on the Atlantic slope of Costa Rica, Central America, indicated that air humidity is maximum at mid-elevations (1900 m), with a dramatic fall to the lowlands and just a minor decline to higher elevations (Kluge et al., 2006). However, moisture is most likely restricting fern richness in the lowlands because high temperatures induce increased evaporation potential and hence water stress (Zotz and Hietz, 2001).

### Relationship of fern richness to rainfall

The relationship of fern species richness to rainfall follows the same pattern as that for moisture availability. Fern species richness along the MAP gradient displays a log-linear trend (**Figure 5S**). According to Tang, Li, Li and Meng (Tang et al., 2014), MAP was a good indicator to predict fern richness in both multiple and linear regression models, with a significant correlation with fern species richness (**Figure 5**). Furthermore, the MAP peak occurred at a mid-elevation region in China’s Taibai Mountain, which is readily to generate a cloud zone, allowing a huge amount of water to be deposited directly onto plants from light mist and clouds, resulting in the development of plant growth development. The largest MAP (784 mm) for species richness was reported in our study at 2500 m, which might be attributed to the high fern richness at TP’s mid-elevation regions. The elevational trends of rainfall and the cloud cover duration, varies greatly over the region (Vetaas et al., 2019). It rises from the lowlands to an elevational belt between 800 and 2000 m, but then falls at higher altitudes (Acharya et al., 2011). According to Stevens (1989), the creation of plant elevational richness patterns is highly related to rainfall patterns.

Fern richness declined less dramatically at higher elevations occupied by open alpine habitats than at lower elevations (Anderson, 2018). This is most likely due to extremely low precipitation at higher altitudes (**Figure 4D**). During the rest of the year, the moisture is locked up as snow, making it mostly inaccessible to plants. Nevertheless, the significant decrease in species richness reported above 2500 m on TP may be attributed in part to eco-physiological factors such as a shorter growing season, low precipitation and ecosystem productivity, high solar radiation in higher altitudes (Körner, 1998). At low elevations, low moisture availability (both precipitation and air humidity) resulted in the decline of fern species (Kessler et al., 2011). Tang, Li, Li and Meng (Tang et al., 2014) observed that low moisture availability restricted species growth at low elevation in China’s Taibai Mountain. The lowest richness of fern species was seen in lowland disturbed areas that were relatively dry (Marini et al., 2011).

### Relationship of fern richness and solar radiation

The sunshine percentage (an indicator of the solar radiation) had a significant positive correlation (0.86) with elevation and significant negative correlation (-0.43) with fern richness (**Figure 5**). According to Barry (2008), solar radiation showed an increasing trend with elevation gradient as shown in **Figure 4**. With rising sunshine, the richness of fern species should decrease linearly. There is no pattern below 2000 m, however there is a positive tendency above 2000 m. This is most likely due to very strong solar radiation at higher altitudes (**Figure 4F**), where there is also strong ultra-radiation, dry weather, frequent winds, and low nocturnal temperatures, which may be unfavorable for many ferns (Pouteau et al., 2016). The strong effect of solar radiation and a lack of tree shade above the forest ecotone may cause a decline in fern richness due to higher water evaporation from plant surfaces, resulting in significant water losses (Körner, 2007). Indeed, the life cycles of ferns lead to inadequate regulation of evaporative potential to compensate for excessive levels of water loss (Page, 2002). It is challenging to overcome these stress conditions at higher altitudes with open alpine ecosystems when precipitation and temperature are incredibly low.

### Conservational aspects of fern species along the elevation gradients

Twenty-two of the total species are reported as nearly threatened, vulnerable or critically endangered, and varied in elevation from 800 m to 4200 m (**Figure 4S**). According to Chen, Kay Khine, Yang and Schneider (Chen et al., 2022), the high percentage of endemic (10.7%) and endangered (11.4%) species emphasizes the importance of the region for the preservation of the variety of ferns and lycophytes in China and throughout the world. On the southeast coast of TP, ongoing timber exploitation from low-elevation forest is predicted to have an adverse effect on the fern species (which grows in moist shady places) (Li et al., 2019). Rapoport’s rule (Rapoport, 1982) states that species at lower elevations (tropical zones) have smaller geographical ranges than species in higher elevations (temperate zones) and hence face a higher rate of extinction (Bach et al., 2007). Moreover, temperate species can survive in a variety of environmental conditions due to their adaption to the substantial annual climate change. In addition, the adaptation of temperate species to the strong annual climatic variation allows for survival across a wide range of environmental conditions (Stevens, 1989).

Reduced plant cover and cultivation of steeper slopes with lower canopy cover crops has led in soil erosion and degradation (Guerra et al., 2020), both of which can amplify the effects of land use and climate change (Paustian et al., 2016; Vijith and Dodge-Wan, 2018) and worsen the status of local biodiversity (Wall et al., 2015). According to Cicuzza and Mammides (2022), soil qualities and slope have an impact on fern richness and abundance, particularly in the steep lowland terrain of Xishuangbanna, where most of the natural forest has been replaced by rubber plantations in recent decades. The abundance of ferns declines dramatically as well, from 76.5% to 26.0% on the eastern TP, which may be linked to considerable changes in local hydrological conditions caused by human and natural disturbance (Wei et al., 2021). The significance of soil and slope to the diversity and abundance of fern species highlights how disturbed sites, such as those with declining tree populations and canopy cover, may change the characteristics of the soil, which has a detrimental effect on fern species diversity and the overall productivity of the ecosystem.

Ferns have faced a new type of threat over the last five decades, as humans have damaged natural ecosystems and caused dramatic species extinction (Pimm and Raven, 2000). Ferns that can adapt to fire, human disturbance, and agricultural contexts may grow, while ferns in undisturbed ecosystems such as lowland and montane tropical forests face extinction. Ferns are of particular interest in biodiversity conservation because they are limited to a small geographical range and are susceptible to loss due to timber harvesting, habitat loss, and degradation (Mehltreter, 2010). Further scientific research is needed to address the impact of forest loss, habitat degradation, and habitat loss on the altitudinal distribution of fern richness at intermediate elevations in this region (Liang et al., 2020). Considering land-use constraints and climate change, the recognition of elevational boundaries with high concentrations of critically endangered fern species is useful for strategic conservation planning.

## Conclusion

In conclusion, the findings provided here have (1): demonstrated strong associations between fern species richness and climatic factors along the altitudinal gradient (2) enabled us to completely negate the claim that fern species has a monotonically increasing trend along the altitudinal gradient and altered it with an effective unimodal concept (3), supported idea that the fern species showed similar pattern to from other parts of the regions. At the mid-elevation peak, an ideal range of temperature and precipitation (high moisture availability) may result in increased energy availability and, as a result, higher species richness. At the lower elevation, high temperature and low precipitation resulted in low moisture availability, consequently, restrict the ferns growth, whereas at higher elevation, species richness was limited by high solar radiation. Nevertheless, because these assumptions are based on extrapolated species and climatic data, they must be validated by true sampling from established sample plots with measurable climatic variables. Further scientific research is required to explore the influence of forest degradation and habitat loss on the altitudinal distribution of fern richness in this region at intermediate altitudes.

## Data availability statement

The original contributions presented in the study are included in the article/**Supplementary Material**. Further inquiries can be directed to the corresponding authors.

## Author contributions

JN and MU designed the study. XH, QC, SA and MU prepared the species distribution data and climate data. All authors were involved in data analysis and interpolation. MU prepared the first draft and JN revised the manuscript. All authors contributed to the article and approved the submitted version.
