# Supplementary material for: Distribution patterns of fern species richness along elevations the Tibetan Plateau in China: regional differences and effects of climate change variables
Source: Front Plant Sci. 2023 May 9;14:1178603. doi: 10.3389/fpls.2023.1178603 (PMC10203567; doi:10.3389/fpls.2023.1178603)
Supplement: Supplementary file 1 [file DataSheet_1.docx]

**Supplementary Figures**





Figure S1. Map of the study area


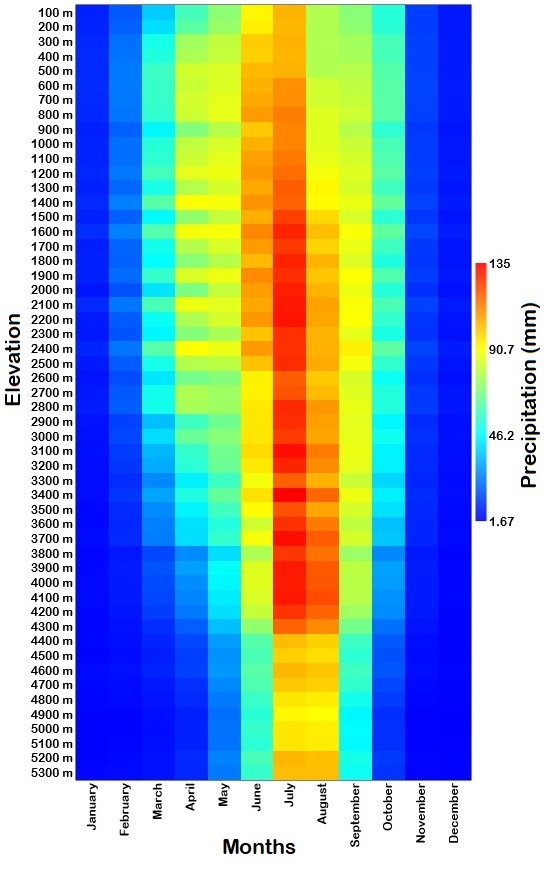

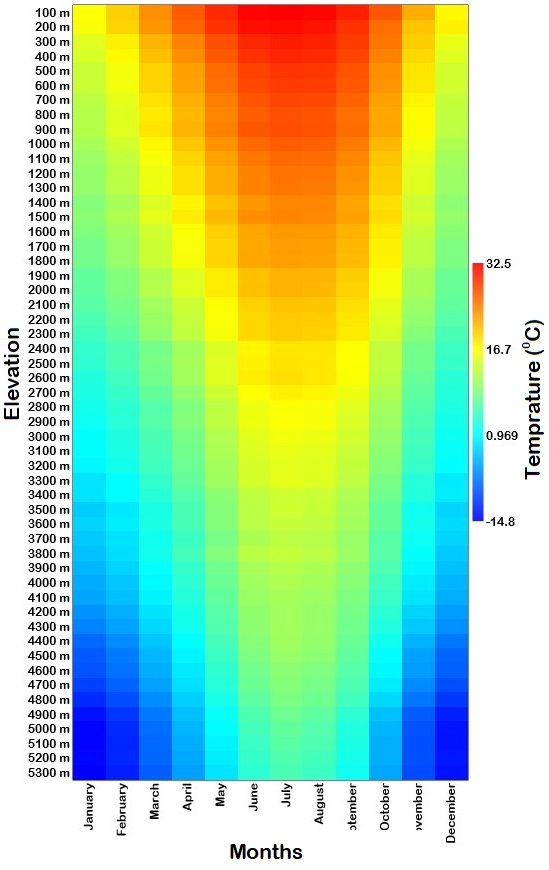

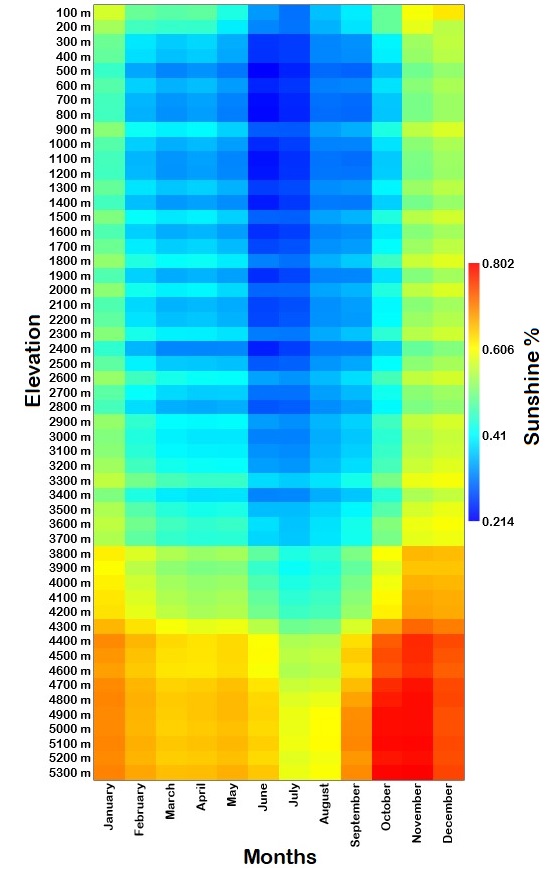


**Figure S2.** Heatmap showing the relationship between elevation (m) and monthly data of climatic variables i.e., (a) precipitation (%), (b) temperature (^0^C) and (c) sunshine (%) in the Tibetan Plateau, China.


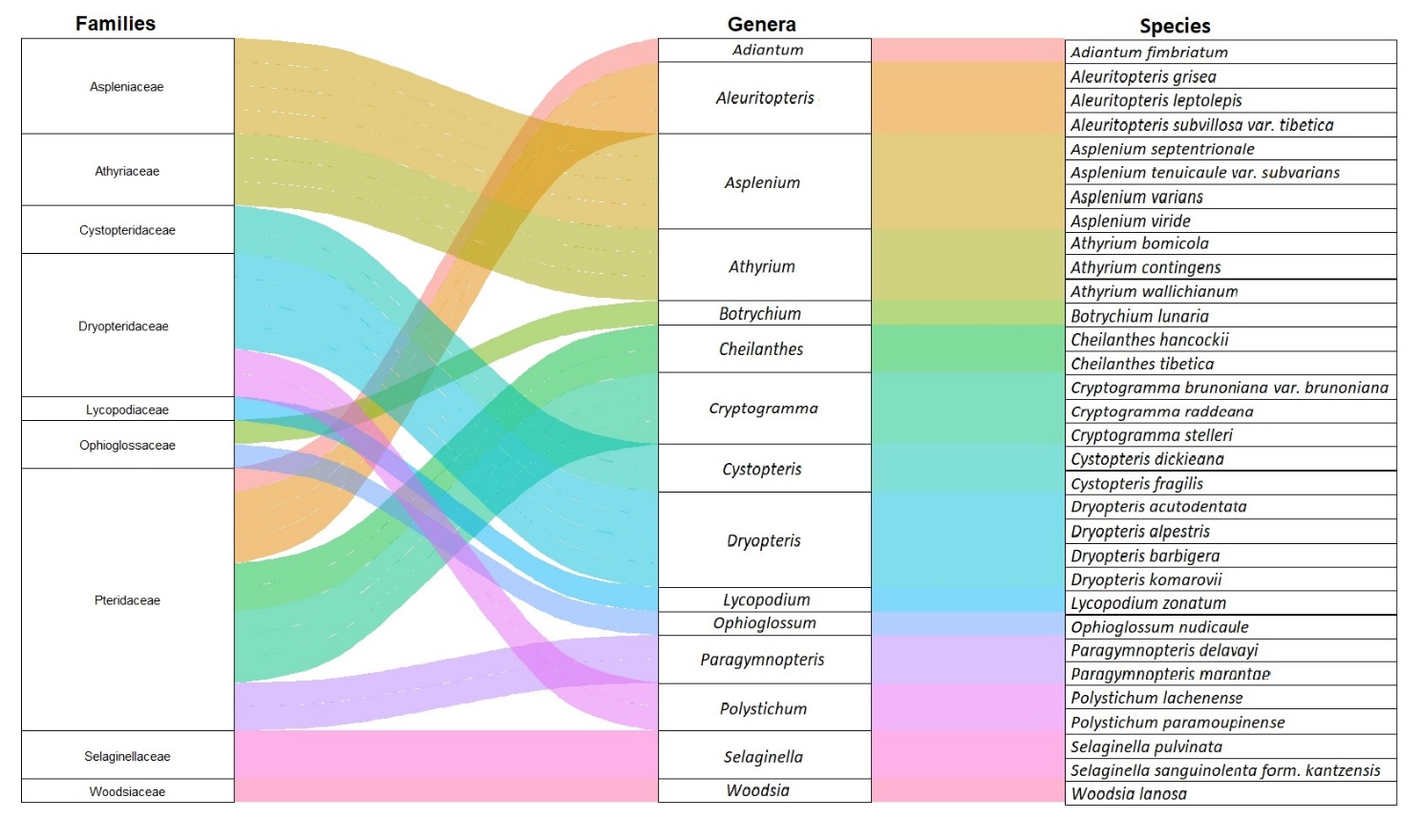


**Figure S3.** Alluvial plots showing the fern species richness above 4000-5300 m a.s.l.


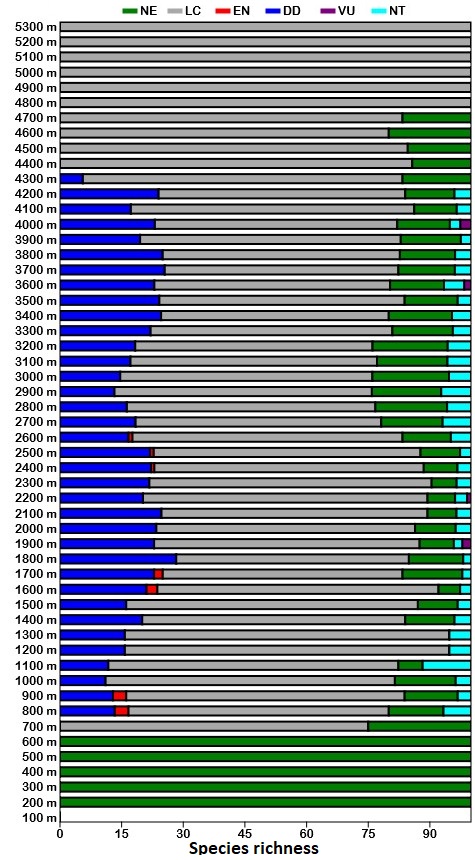


**Figure S4** The conservation status of fern species along the elevations on Tibetan Plateau, China.
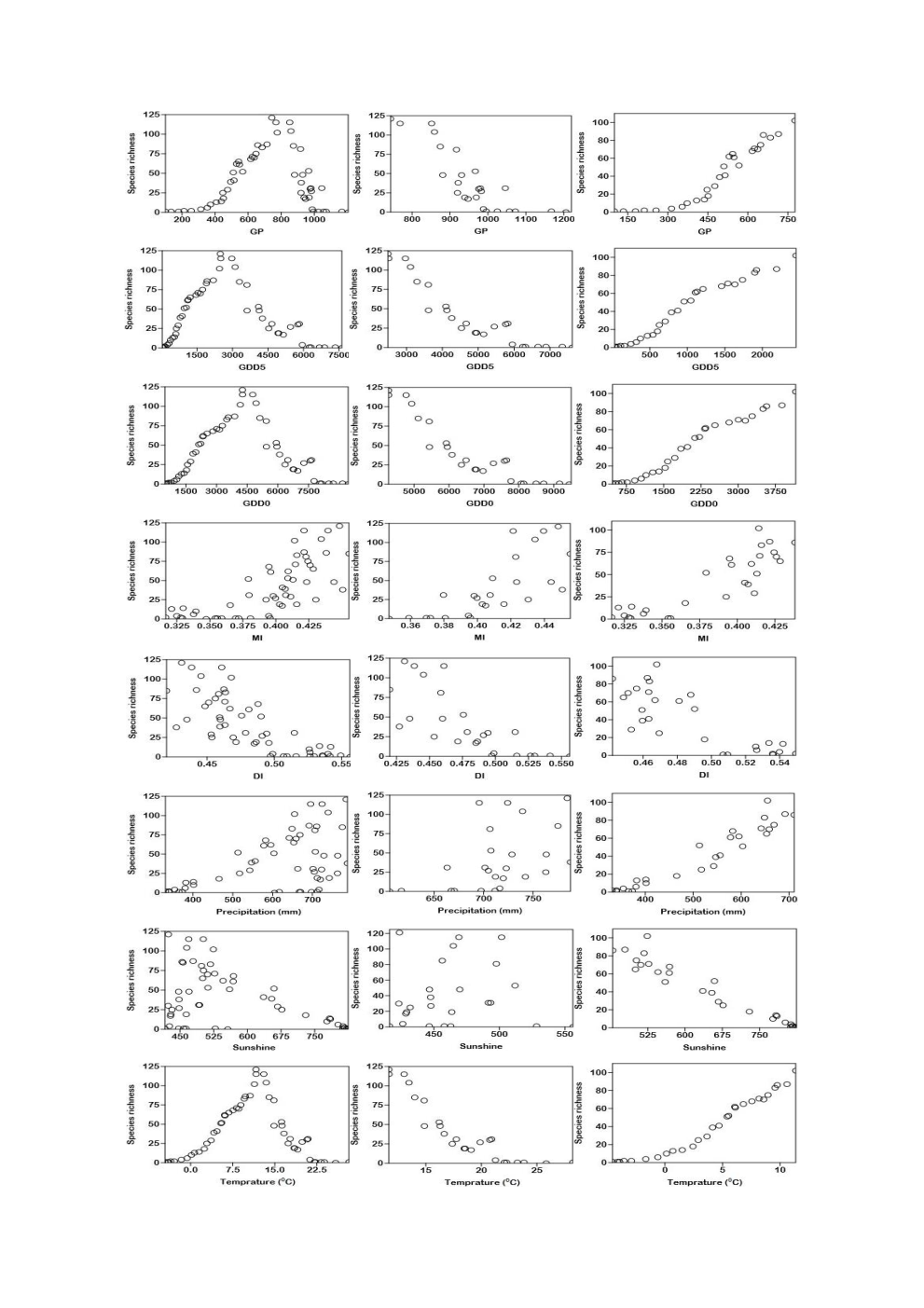


**Figure S5.** Scatter plots showing the relationship between species richness (y-axis), and different climatic variables i.e., (a) GP, (b) GDD_0_, (c) GDD_5_, (d) MI, (e) DI, (f) SS%, (g) MAP, and (h) MAT (^0^C) (x-axis): (I) whole elevation gradient 100-4800 m a.s.l. (II) low elevation sub-gradient 100-2500 m a.s.l. (III) upper sub-gradient 2500-5300 m a.s.l on Tibetan Plateau, China.
