# Supplementary material for: Distribution patterns of fern species richness along elevations the Tibetan Plateau in China: regional differences and effects of climate change variables
Source: Front Plant Sci. 2023 May 9;14:1178603. doi: 10.3389/fpls.2023.1178603 (PMC10203567; doi:10.3389/fpls.2023.1178603)
Supplement: Supplementary file 2 [file Table_1.docx]

**Supplementary Table**

**Supplementary Table 1. Regional distribution of fern species in Xizang Tibet, China.**

| **Family** | **Species** | **Status** | **Elevation (m)** |
| --- | --- | --- | --- |
| Aspidiaceae | *Dryopteris apiciflora* | NE | 2200~3300 |
|  | *Dryopteris clarkei* | NE | 2500~2800 |
|  | *Dryopteris heterolaena* | NE | 1400~2300 |
|  | *Dryopteris transmorrisonense* | NE | 2600~3300 |
|  | *Tectaria polymorpha* | LC | 800 |
| Aspleniaceae | [*Asplenium adiantum-nigrum*](https://www.plantplus.cn/info/Asplenium%20adiantum-nigrum) | DD | 2500~2700 |
|  | [*Hymenasplenium cheilosorum*](https://www.plantplus.cn/info/Hymenasplenium%20cheilosorum) | LC | 900 |
|  | *Asplenium ensiforme* | LC | 2200~2650 |
|  | *Asplenium scortechinii* | LC | 1700~2800 |
|  | [*Hymenasplenium excisum*](https://www.plantplus.cn/info/Hymenasplenium%20excisum) | LC | 850 |
|  | *Asplenium finlaysonianum* | LC | 900 |
|  | *Asplenium griffithianum* | LC | 850 |
|  | *Asplenium laciniatum var. acutipinna* | NE | 1800 |
|  | *Asplenium nesii* | LC | 3300 |
|  | *Asplenium normale* | LC | 2200 |
|  | *Asplenium prolongatum* | LC | 900~2000 |
|  | *Asplenium ruta-muraria* | LC | 3300 |
|  | *Asplenium septentrionale* | LC | 4000~4100 |
|  | *Asplenium tenuicaule var. subvarians* | LC | 2900~4100 |
|  | *Asplenium tenuifolium* | LC | 2000~2400 |
|  | *Asplenium pseudo-fontanum* | DD | 2500 |
|  | *Asplenium trichomanes* | LC | 2300~4000 |
|  | *Asplenium varians* | LC | 2200~4600 |
|  | [*Asplenium viride*](https://www.plantplus.cn/info/Asplenium%20viride) | LC | 4100 |
|  | [*Asplenium indicum*](https://www.plantplus.cn/info/Asplenium%20indicum) | LC | 1800~2300 |
|  | [*Asplenium exiguum*](https://www.plantplus.cn/info/Asplenium%20exiguum) | LC | 2380 |
|  | [*Asplenium paucivenosum*](https://www.plantplus.cn/info/Asplenium%20paucivenosum) | DD | 2000~2700 |
|  | [*Asplenium phyllitidis*](https://www.plantplus.cn/info/Asplenium%20phyllitidis) | NE | 800~1000 |
| Athyriaceae | [*Diplazium axillare*](https://www.plantplus.cn/info/Diplazium%20axillare) | LC | 2000~2600 |
|  | *Diplazium hirsutipes* | LC | 1900~2600 |
|  | *Diplazium laxifrons* | LC | 1500~2080 |
|  | [*Diplazium latipinnulum*](https://www.plantplus.cn/info/Diplazium%20latipinnulum) | DD | 800 |
|  | [*Diplazium lobulosum*](https://www.plantplus.cn/info/Diplazium%20lobulosum) | DD | 1800 |
|  | *Diplazium medogensis* | DD | 1900 |
|  | [*Diplazium squamigerum*](https://www.plantplus.cn/info/Diplazium%20squamigerum) | LC | 2500 |
|  | *Diplazium subspectabilis* | DD | 2200 |
|  | *Diplazium tibeticum* | DD | 1700 |
|  | *Diplazium viridissimum* | LC | 1800~2200 |
|  | *Athyrium anisopterum* | LC | 2000 |
|  | *Athyrium austro-orientale* | NE | 2400~3800 |
|  | *Athyrium foliolosum* | NE | 1800~1880 |
|  | *Athyrium schimperi* | LC | 2200~3000 |
|  | *Athyrium bomicola* | DD | 4100 |
|  | *Athyrium bucahwangense* | NE | 2600~3800 |
|  | *Athyrium contingens* | DD | 3100~4200 |
|  | *Athyrium delicatulum* | LC | 1530 |
|  | *Athyrium dentilobum* | DD | 2600 |
|  | *Athyrium drepanopterum* | DD | 1500~2200 |
|  | *Athyrium dubium* | DD | 3600~3700 |
|  | *Athyrium davidii* | DD | 3000 |
|  | *Athyrium filix-femina* | NE | 3400~3900 |
|  | *Athyrium fimbriatum* | DD | 2400~2800 |
|  | *Athyrium flabellulatum* | DD | 3700 |
|  | *Athyrium iseanum* | LC | 2800 |
|  | [*Athyrium mackinnonorum*](https://www.plantplus.cn/info/Athyrium%20mackinnonorum) | LC | 2200 |
|  | *Athyrium mackinnonii var. glabratum* | NE | 2400 |
|  | *Athyrium mackinnonii var. yigongense* | NE | 2380 |
|  | *Athyrium medogense* | DD | 2200 |
|  | *Athyrium nakanoi* | DD | 1800~2200 |
|  | *Athyrium nigripes* | LC | 2000 |
|  | *Athyrium nyalamense* | DD | 2200~2600 |
|  | *Athyrium pectinatum* | DD | 2100 |
|  | *Athyrium rhachidosorum* | DD | 1800~2400 |
|  | *Athyrium rupicola* | DD | 3200~3800 |
|  | *Athyrium mehrae* | DD | 3250 |
|  | *Athyrium strigillosum* | LC | 1800~2600 |
|  | *Athyrium foliolosum* | NE | 1700 |
|  | *Athyrium dentigerum* | LC | 2000~3900 |
|  | *Athyrium wallichianum* | LC | 3900~4250 |
|  | *Athyrium zayuense* | DD | 3350 |
|  | *Cornopteris latiloba* | DD | 2400 |
|  | [*Deparia boryana*](https://www.plantplus.cn/info/Deparia%20boryana) | LC | 3300 |
|  | [*Anisocampium cuspidatum*](https://www.plantplus.cn/info/Anisocampium%20cuspidatum) | VU | 1850 |
|  | *Deparia acuta var. bagaensis* | NE | 3400 |
|  | *Deparia acuta* | NE | 3400~4000 |
|  | [*Deparia medogensis*](https://www.plantplus.cn/info/Deparia%20medogensis) | DD | 2800 |
|  | [*Deparia sichuanensis*](https://www.plantplus.cn/info/Deparia%20sichuanensis) | LC | 1400-3500 |
|  | [*Deparia sikkimensis*](https://www.plantplus.cn/info/Deparia%20sikkimensis) | DD | 3000~3800 |
|  | [*Deparia wilsonii*](https://www.plantplus.cn/info/Deparia%20wilsonii) | DD | 2800~3500 |
|  | [*Deparia wilsonii var. muliensis*](https://www.plantplus.cn/info/Deparia%20wilsonii%20var.%20muliensis) | DD | 3300~3800 |
|  | [*Athyrium atkinsonii*](https://www.plantplus.cn/info/Athyrium%20atkinsonii) | LC | 3300 |
|  | [*Athyrium schizochlamys*](https://www.plantplus.cn/info/Athyrium%20schizochlamys) | LC | 3100 |
| Blechnaceae | [*Blechnopsis orientalis*](https://www.plantplus.cn/info/Blechnopsis%20orientalis) | LC | 800 |
|  | [*Woodwardia unigemmata*](https://www.plantplus.cn/info/Woodwardia%20unigemmata) | NE | 2000~3000 |
| Cibotiaceae | *Cibotium barometz* | LC | 900 |
| Cyatheaceae | *Alsophila spinulosa* | NT | 1060 |
|  | [*Gymnosphaera andersonii*](https://www.plantplus.cn/info/Gymnosphaera%20andersonii) | EN | 800 |
|  | *Sphaeropteris brunoniana* | EN | 850 |
| Cystopteridaceae | *Acystopteris tenuisecta* | LC | 2000 |
|  | *Cystopteris dickieana* | LC | 3600~5400 |
|  | *Cystopteris fragilis* | LC | 3800~4800 |
|  | *Cystopteris kansuana* | LC | 3800 |
|  | *Cystopteris montana* | LC | 3700~3950 |
|  | *Cystopteris moupinensis* | LC | 2500~3700 |
|  | *Cystopteris pellucida* | LC | 3600 |
|  | *Cystopteris sudetica* | LC | 3500 |
|  | *Cystopteris tibetica* | DD | 2400-3600 |
|  | *Gymnocarpium remotepinnatum* | LC | 2600~3100 |
|  | *Gymnocarpium oyamense* | LC | 2200 |
| [Davalliaceae](https://www.iplant.cn/info/Pteridaceae) | [*Davallia pulchra*](https://www.plantplus.cn/info/Davallia%20pulchra) | NE | 2700~3500 |
|  | *Araiostegia faberiana* | LC | 3000 |
|  | [*Davallia hookeri*](https://www.plantplus.cn/info/Davallia%20hookeri) | DD | 3100~3400 |
|  | [*Davallia perdurans*](https://www.plantplus.cn/info/Davallia%20perdurans) | LC | 2200~2300 |
|  | [*Davallia assamica*](https://www.plantplus.cn/info/Davallia%20assamica) | DD | 1700~2300 |
|  | [*Davallia griffithiana*](https://www.plantplus.cn/info/Davallia%20griffithiana) | DD | 1600~2200 |
| [Dennstaedtiaceae](https://www.iplant.cn/info/Pteridaceae) | *Dennstaedtia scabra* | LC | 1500 |
|  | *Dennstaedtia appendiculata* | DD | 2300~2400 |
|  | *Dennstaedtia elwesii* | DD | 2400 |
|  | *Microlepia khasiyana* | LC | 850 |
|  | *Microlepia platyphylla* | LC | 2200 |
|  | *Microlepia trapeziformis* | LC | 750 |
|  | *Pteridium aquilinum var. latiusculum* | LC | 2700~3100 |
|  | *Pteridium revolutum* | LC | 1200~3100 |
|  | *Histiopteris incisa* | LC | 2200~2400 |
| [Dipteridaceae](https://www.iplant.cn/info/Pteridaceae) | *Dipteris wallichii* | DD | 1400 |
| [Dryopteridaceae](https://www.iplant.cn/info/Cystopteridaceae) | *Bolbitis tibetica* | DD | 800 |
|  | *Bolbitis medogensis* | DD | 900 |
|  | *Lomagramma grosseserrata* | NE | 800 |
|  | [*Elaphoglossum marginatum*](https://www.plantplus.cn/info/Elaphoglossum%20marginatum) | LC | 2400~2600 |
|  | [*Dryopteris paleolata*](https://www.plantplus.cn/info/Dryopteris%20paleolata) | LC | 2600 |
|  | [*Dryopteris hookeriana*](https://www.plantplus.cn/info/Dryopteris%20hookeriana) | DD | 1700~2200 |
|  | *Dryopteris annamensis* | NE | 1700 |
|  | *Dryopteris medogensis* | NE | 1800 |
|  | [*Dryopteris peranema*](https://www.plantplus.cn/info/Dryopteris%20peranema) | LC | 2380 |
|  | [*Dryopteris diffracta*](https://www.plantplus.cn/info/Dryopteris%20diffracta) | LC | 1700 |
|  | *Arachniodes simplicior* | LC | 1600 |
|  | *Cyrtomium caryotideum* | LC | 2000~2450 |
|  | *Cyrtomium lonchitoides* | LC | 2400~2600 |
|  | *Cyrtomium macrophyllum* | NE | 2000 |
|  | *Dryopteris acutodentata* | DD | 3500~4150 |
|  | *Dryopteris alpestris* | DD | 3700~4200 |
|  | *Dryopteris barbigera* | LC | 3600~4700 |
|  | *Dryopteris blanfordii* | LC | 2900~3500 |
|  | *Dryopteris chrysocoma* | NE | 2700~3200 |
|  | *Dryopteris atrata* | LC | 2300~2500 |
|  | *Dryopteris fibrillosissima* | NE | 2280 |
|  | *Dryopteris fructuosa* | LC | 2200~2700 |
|  | *Dryopteris stenolepis* | LC | 2200 |
|  | *Dryopteris blanfordii subsp. nigrosquamosa* | NE | 2880~3500 |
|  | *Dryopteris juxtaposita* | LC | 2200~2300 |
|  | *Dryopteris lachoongensis* | VU | 2200 |
|  | *Dryopteris lepidopoda* | LC | 2300~2500 |
|  | *Dryopteris nyingchiensis* | NE | 2900~3000 |
|  | *Dryopteris panda* | LC | 1500~2600 |
|  | *Dryopteris redactopinnata* | LC | 3000~3600 |
|  | *Dryopteris sikkimensis* | DD | 2300 |
|  | *Dryopteris pulcherrima* | LC | 2000 |
|  | *Dryopteris scottii* | LC | 1800 |
|  | *Dryopteris serratodentata* | NE | 3400~3880 |
|  | *Dryopteris sinofibrillosa* | LC | 2800~3900 |
|  | *Dryopteris sparsa* | LC | 1400~2000 |
|  | *Dryopteris squamifera* | NE | 3500~3600 |
|  | *Dryopteris komarovii* | LC | 2800~4500 |
|  | *Dryopteris sublacera* | LC | 2000~4000 |
|  | *Dryopteris wallichiana* | LC | 1500~2600 |
|  | *Dryopteris yigongensis* | DD | 2500 |
|  | *Dryopteris woodsiisora* | LC | 3200~3300 |
|  | [*Arachniodes superba*](https://www.plantplus.cn/info/Arachniodes%20superba) | LC | 2500 |
|  | *Polystichum acanthophyllum* | LC | 2300~2800 |
|  | *Polystichum mehrae* | DD | 2400~2500 |
|  | *Polystichum acutidens* | LC | 4000 |
|  | *Polystichum squarrosum* | DD | 2400 |
|  | *Polystichum neolobatum* | LC | 3000 |
|  | *Polystichum atkinsonii* | LC | 2800~2900 |
|  | *Polystichum auriculatum* | NE | 900 |
|  | *Polystichum bakerianum* | DD | 3500~3600 |
|  | *Polystichum piceopaleaceum* | LC | 2400~2650 |
|  | *Polystichum bomiense* | DD | 4000 |
|  | [*Polystichum garhwalicum*](https://www.plantplus.cn/info/Polystichum%20garhwalicum) | LC | 2300~3400 |
|  | *Polystichum pseudocastaneum* | DD | 3500~3700 |
|  | *Polystichum capillipes* | LC | 2900 |
|  | *Polystichum castaneum* | DD | 3250~3300 |
|  | [*Polystichum stenophyllum*](https://www.plantplus.cn/info/Polystichum%20stenophyllum) | DD | 2900 |
|  | *Polystichum deltodon* | LC | 1300~2600 |
|  | *Polystichum discretum* | DD | 2400~2500 |
|  | *Polystichum duthiei* | DD | 2400~2600 |
|  | *Polystichum tsus-simense* | LC | 2070~2300 |
|  | *Polystichum gongboense* | DD | 3000~3500 |
|  | [*Polystichum yunnanense*](https://www.plantplus.cn/info/Polystichum%20yunnanense) | NE | 2450 |
|  | *Polystichum lachenense* | LC | 3800~5000 |
|  | *Polystichum lentum* | DD | 1400 |
|  | *Polystichum longidens* | DD | 4000 |
|  | *Polystichum longipaleatum* | LC | 1900~3100 |
|  | [*Polystichum moupinense*](https://www.plantplus.cn/info/Polystichum%20moupinense) | DD | 4000 |
|  | *Polystichum mollissimum* | DD | 3400 |
|  | *Polystichum microchlamys* | NE | 2600~3400 |
|  | *Polystichum nepalense* | DD | 2300~2800 |
|  | *Polystichum orientalitibeticum* | DD | 2000 |
|  | *Polystichum paramoupinense* | DD | 4150 |
|  | *Polystichum prescottianum* | DD | 3400~4000 |
|  | *Polystichum punctiferum* | DD | 2400~3000 |
|  | *Polystichum qamdoense* | DD | 3600~4100 |
|  | *Polystichum rhombiforme* | DD | 3100~3300 |
|  | *Polystichum semifertile* | DD | 1800 |
|  | *Polystichum shensiense* | DD | 2400~3600 |
|  | *Polystichum stimulans* | DD | 2400~2700 |
|  | *Polystichum thomsonii* | LC | 3200 |
|  | *Polystichum tibeticum* | DD | 3600 |
|  | *Polystichum wattii* | DD | 1700 |
|  | *Polystichum yadongense* | DD | 3800 |
|  | *Polystichum yigongense* | NE | 2400 |
|  | [*Polystichum glaciale*](https://www.plantplus.cn/info/Polystichum%20glaciale) | NT | 3600 |
| [Equisetaceae](https://www.iplant.cn/info/Cystopteridaceae) | *Equisetum arvense* | LC | 3200 |
|  | *Equisetum diffusum* | LC | 800~1900 |
|  | *Equisetum palustre* | LC | 3500~3900 |
|  | *Equisetum ramosissimum subsp. debile* | LC | 1580~3200 |
|  | [*Equisetum ramosissimum*](https://www.plantplus.cn/info/Equisetum%20ramosissimum) | LC | 2300~3600 |
| Gleicheniaceae | *Dicranopteris ampla* | LC | 700~900 |
|  | *Dicranopteris linearis* | LC | 800 |
|  | *Diplopterygium giganteum* | DD | 2300~2800 |
| Hymenophyllaceae | *Crepidomanes latealatum* | NE | 2000 |
|  | *Crepidomanes zayuense* | DD | 2000~2400 |
|  | *Hymenophyllum simonsianum* | DD | 2400 |
|  | [*Hymenophyllum badium*](https://www.plantplus.cn/info/Hymenophyllum%20badium) | LC | 1500~1900 |
|  | [*Hymenophyllum exsertum*](https://www.plantplus.cn/info/Hymenophyllum%20exsertum) | LC | 2200~2500 |
|  | [*Hymenophyllum corrugatum*](https://www.plantplus.cn/info/Hymenophyllum%20corrugatum) | NE | 2400 |
|  | [*Hymenophyllum levingei*](https://www.plantplus.cn/info/Hymenophyllum%20levingei) | DD | 2300 |
|  | [*Hymenophyllum longissimum*](https://www.plantplus.cn/info/Hymenophyllum%20longissimum) | DD | 2400~2900 |
|  | *Hymenophyllum polyanthos* | NE | 2800~3300 |
|  | *Vandenboschia auriculata* | LC | 1700~1800 |
|  | [*Vandenboschia striata*](https://www.plantplus.cn/info/Vandenboschia%20striata) | LC | 900~2100 |
|  | *Trichomanes tibeticum* | DD | 2300 |
| [Hypodematiaceae](https://www.iplant.cn/info/Hypodematiaceae) | *Hypodematium hirsutum* | LC | 2300 |
|  | [*Leucostegia truncata*](https://www.plantplus.cn/info/Leucostegia%20truncata) | DD | 2100~2400 |
| Lindsaeaceae | *Osmolindsaea odorata* | LC | 2400~2900 |
|  | *Odontosoria chinensis* | LC | 780~2200 |
| Lycopodiaceae | *Huperzia bucahwangensis* | DD | 2400 |
|  | *Huperzia herteriana* | DD | 3750 |
|  | *Huperzia lajouensis* | DD | 3000 |
|  | *Phlegmariurus nylamensis* | DD | 1800~2000 |
|  | *Huperzia selago* | VU | 4000 |
|  | *Huperzia appressa* | NE | 2000 |
|  | *Huperzia serrata* | EN | 1530 |
|  | *Phlegmariurus cancellatus* | DD | 2300 |
|  | *Phlegmariurus pulcherrimus* | DD | 1800 |
|  | *Phlegmariurus squarrosus* | NT | 800 |
|  | *Diphasiastrum complanatum* | LC | 1600 |
|  | *Lycopodium zonatum* | DD | 4200 |
|  | *Lycopodium japonicum* | LC | 1300~2700 |
|  | [*Dendrolycopodium obscurum*](https://www.plantplus.cn/info/Dendrolycopodium%20obscurum) | LC | 2600 |
|  | *Palhinhaea cernua* | LC | 800~1300 |
| [Lygodiaceae](https://www.iplant.cn/info/Lygodiaceae) | *Lygodium japonicum* | LC | 700~1600 |
| Marattiaceae | *Angiopteris esculenta* | DD | 1500 |
|  | *Angiopteris wallichiana* | DD | 1800 |
| Nephrolepidaceae | *Nephrolepis cordifolia* | LC | 850~1700 |
| Oleandraceae | *Oleandra wallichii* | LC | 1750~2600 |
| Onocleaceae | [*Pentarhizidium orientale*](https://www.plantplus.cn/info/Pentarhizidium%20orientale) | LC | 2050 |
|  | *Matteuccia struthiopteris* | LC | 2800~3000 |
| Ophioglossaceae | *Botrychium lunaria* | LC | 3100~4100 |
|  | [*Japanobotrychum lanuginosum*](https://www.plantplus.cn/info/Japanobotrychum%20lanuginosum) | NT | 2000~3100 |
|  | [*Botrypus virginianus*](https://www.plantplus.cn/info/Botrypus%20virginianus) | LC | 2200~2900 |
|  | *Ophioglossum nudicaule* | DD | 3100~4300 |
|  | *Ophioglossum reticulatum* | NT | 2250~2900 |
|  | *Ophioglossum vulgatum* | LC | 3100 |
| [Osmundaceae](https://www.google.com/search?hl=en&biw=1920&bih=937&sxsrf=ALiCzsYjaXDFqqTiW7lBseTWW8ErpEtRgw:1658456249012&q=Osmundaceae&stick=H4sIAAAAAAAAAONgVuLUz9U3MLQsSyp5xGjCLfDyxz1hKe1Ja05eY1Tl4grOyC93zSvJLKkUEudig7J4pbi5ELp4FrFy-xfnlualJCanJqYCAGUpjWpSAAAA&sa=X&ved=2ahUKEwiv6OP8tov5AhXdK0QIHVt2A6QQzIcDKAB6BAgMEAE) | *Osmunda claytoniana* | LC | 2200~3600 |
|  | [*Claytosmunda claytoniana*](https://www.plantplus.cn/info/Claytosmunda%20claytoniana) | LC | 800~1800 |
| Plagiogyriaceae | *Plagiogyria pycnophylla* | LC | 2100~2300 |
| Plagiogyriaceae | [*Plagiogyria glauca*](https://www.plantplus.cn/info/Plagiogyria%20glauca) | NE | 2400~2800 |
| Polypodiaceae | [*Selliguea dareiformis*](https://www.plantplus.cn/info/Selliguea%20dareiformis) | EN | 2400~2600 |
|  | [*Selliguea himalayensis*](https://www.plantplus.cn/info/Selliguea%20himalayensis) | DD | 2000~2500 |
|  | [*Selliguea intermedia*](https://www.plantplus.cn/info/Selliguea%20intermedia) | DD | 2000 |
|  | [*Selliguea lehmannii*](https://www.plantplus.cn/info/Selliguea%20lehmannii) | LC | 2400 |
|  | [*Selliguea mairei*](https://www.plantplus.cn/info/Selliguea%20mairei) | LC | 2600 |
|  | [*Selliguea capitellata*](https://www.plantplus.cn/info/Selliguea%20capitellata) | LC | 2100~2400 |
|  | [*Selliguea wardii*](https://www.plantplus.cn/info/Selliguea%20wardii) | LC | 1500~2400 |
|  | [*Leptochilus hemionitideus*](https://www.plantplus.cn/info/Leptochilus%20hemionitideus) | LC | 850 |
|  | [*Lepisorus miyoshianus*](https://www.plantplus.cn/info/Lepisorus%20miyoshianus) | LC | 2100~2500 |
|  | [*Lepisorus rostratus*](https://www.plantplus.cn/info/Lepisorus%20rostratus) | LC | 2040 |
|  | *Lepisorus bicolor* | LC | 2800 |
|  | *Lepisorus clathratus* | LC | 3400~3700 |
|  | *Lepisorus contortus* | LC | 2300~3500 |
|  | [*Lepisorus nudus*](https://www.plantplus.cn/info/Lepisorus%20nudus) | DD | 2200~2380 |
|  | *Lepisorus lineariformis* | NE | 1750 |
|  | *Lepisorus loriformis* | LC | 2000~2900 |
|  | *Lepisorus stenistus* | LC | 2500~2950 |
|  | *Lepisorus macrosphaerus* | LC | 2000~2600 |
|  | *Lepisorus morrisonensis* | LC | 2000~3600 |
|  | *Lepisorus sordidus* | DD | 2500 |
|  | *Lepisorus oligolepidus* | LC | 2300 |
|  | *Lepisorus tibeticus* | LC | 2400~2500 |
|  | *Lepisorus pseudonudus* | LC | 3400 |
|  | *Lepisorus scolopendrium* | LC | 2000~2800 |
|  | *Lepisorus eilophyllus* | LC | 3800 |
|  | *Microsorum insigne* | LC | 800 |
|  | *Microsorium excelgum* | NE | 1620 |
|  | [*Lepisorus fortunei*](https://www.plantplus.cn/info/Lepisorus%20fortunei) | LC | 1200~2600 |
|  | [*Bosmania membranacea*](https://www.plantplus.cn/info/Bosmania%20membranacea) | NE | 2100 |
|  | [*Lepisorus superficialis*](https://www.plantplus.cn/info/Lepisorus%20superficialis) | NE | 1700 |
|  | [*Lepisorus zippelii*](https://www.plantplus.cn/info/Lepisorus%20zippelii) | NE | 1400~1500 |
|  | [*Lepisorus ovatus*](https://www.plantplus.cn/info/Lepisorus%20ovatus) | NE | 200~1100 |
|  | [*Microsorum cuspidatum*](https://www.plantplus.cn/info/Microsorum%20cuspidatum) | LC | 900 |
|  | [*Selliguea malacodon*](https://www.plantplus.cn/info/Selliguea%20malacodon) | NE | 3200 |
|  | [*Selliguea crenatopinnata*](https://www.plantplus.cn/info/Selliguea%20crenatopinnata) | LC | 2000 |
|  | *Selliguea ebenipes* | LC | 2700~3500 |
|  | [*Selliguea erythrocarpa*](https://www.plantplus.cn/info/Selliguea%20erythrocarpa) | NT | 2700~2800 |
|  | *Selliguea griffithiana* | LC | 2400~2500 |
|  | [*Selliguea albidoglauca*](https://www.plantplus.cn/info/Selliguea%20albidoglauca) | LC | 2400~3700 |
|  | [*Selliguea senanensis*](https://www.plantplus.cn/info/Selliguea%20senanensis) | LC | 3600 |
|  | *Selliguea hastata* | LC | 2000 |
|  | *Selliguea stewartii* | LC | 2400~2700 |
|  | [*Selliguea stracheyi*](https://www.plantplus.cn/info/Selliguea%20stracheyi) | LC | 2800~3200 |
|  | *Selliguea tibetana* | NE | 2400~3400 |
|  | [*Lepisorus waltonii*](https://www.plantplus.cn/info/Lepisorus%20waltonii) | DD | 3400~3900 |
|  | *Lepisorus sinuata* | DD | 2750 |
|  | [*Goniophlebium argutum*](https://www.plantplus.cn/info/Goniophlebium%20argutum) | LC | 2300~2700 |
|  | [*Goniophlebium amoenum*](https://www.plantplus.cn/info/Goniophlebium%20amoenum) | LC | 800~2400 |
|  | *Goniophlebium hendersonii* | DD | 2000~3000 |
|  | *Goniophlebium lachnopus* | LC | 2100 |
|  | *Goniophlebium microrhizoma* | LC | 2400~3200 |
|  | [*Goniophlebium amoenum var. pilosum*](https://www.plantplus.cn/info/Goniophlebium%20amoenum%20var.%20pilosum) | LC | 800~2700 |
|  | [*Goniophlebium subamoenum*](https://www.plantplus.cn/info/Goniophlebium%20subamoenum) | LC | 3000 |
|  | [*Goniophlebium niponicum*](https://www.plantplus.cn/info/Goniophlebium%20niponicum) | DD | 2100~2300 |
|  | *Pyrrosia costata* | NE | 1000 |
|  | *Pyrrosia drakeana* | LC | 2000~2500 |
|  | *Pyrrosia boothii* | DD | 1650 |
|  | [*Pyrrosia davidii*](https://www.plantplus.cn/info/Pyrrosia%20davidii) | LC | 1700~2800 |
|  | *Pyrrosia heteractis* | NE | 1650~2200 |
|  | *Pyrrosia lanceolata* | LC | 800 |
|  | *Pyrrosia stenophylla* | DD | 2300 |
|  | *Pyrrosia lingua* | LC | 780 |
|  | *Pyrrosia porosa var. porosa* | LC | 2000~2500 |
|  | *Pyrrosia stigmosa* | NE | 750 |
|  | *Pyrrosia subfurfuracea* | LC | 1000 |
|  | *Lepisorus normalis* | DD | 800~2200 |
|  | *Drynaria mollis* | NT | 2600 |
|  | *Drynaria delavayi* | VU | 3600 |
|  | *Drynaria propinqua* | NT | 800~2400 |
|  | *Drynaria sinica* | LC | 2600~3800 |
|  | *Drynaria baronii* | LC | 2200~3500 |
|  | [*Drynaria coronans*](https://www.plantplus.cn/info/Drynaria%20coronans) | LC | 2200~2400 |
|  | *Micropolypodium sikkimense* | LC | 2500 |
|  | *Ctenopteris subfalcata* | NT | 2700 |
|  | *Loxogramme chinensis* | LC | 2100~2400 |
|  | *Loxogramme grammitoides* | LC | 1960 |
|  | *Loxogramme involuta* | DD | 2000~2300 |
|  | *Loxogramme lankokiensis* | LC | 900 |
|  | *Loxogramme duclouxii* | LC | 2100~2500 |
|  | *Loxogramme cuspidata* | DD | 2500 |
| Pteridaceae | *Pteris amoena* | LC | 1600 |
|  | *Pteris aspericaulis* | DD | 1800~2400 |
|  | *Pteris aspericaulis var. cuspigera* | DD | 1200~2300 |
|  | *Pteris bomiensis* | DD | 2080 |
|  | *Pteris cretica var. laeta* | LC | 1700~2300 |
|  | *Pteris dactylina* | LC | 2000~3900 |
|  | *Pteris terminalis* | LC | 1680~2400 |
|  | *Pteris fauriei* | LC | 1000 |
|  | *Pteris gracillima* | DD | 800 |
|  | *Pteris medogensis* | DD | 1600 |
|  | *Pteris cretica* | LC | 2000~2600 |
|  | *Pteris pseudodactylina* | DD | 2500 |
|  | *Pteris puberula* | DD | 2700~2900 |
|  | *Pteris vittata* | LC | 800~2600 |
|  | *Pteris wallichiana* | LC | 2100~2600 |
|  | *Aleuritopteris albomarginata* | NT | 2000~2380 |
|  | *Aleuritopteris anceps* | LC | 2200 |
|  | *Aleuritopteris argentea* | LC | 2300~3900 |
|  | *Aleuritopteris chrysophylla* | NT | 1000 |
|  | *Aleuritopteris grisea* | LC | 3200~4600 |
|  | *Aleuritopteris pygmaea* | DD | 3800 |
|  | *Aleuritopteris speciosa* | DD | 3030 |
|  | [*Cheilanthes hancockii*](https://www.plantplus.cn/info/Cheilanthes%20hancockii) | LC | 1750~4110 |
|  | *Cheilanthes insignis* | NE | 2200~3300 |
|  | *Cryptogramma brunoniana var. brunoniana* | LC | 3300~4360 |
|  | *Cryptogramma raddeana* | NE | 3600~4700 |
|  | *Cryptogramma stelleri* | LC | 3600~4700 |
|  | [*Aleuritopteris kuhnii*](https://www.plantplus.cn/info/Aleuritopteris%20kuhnii) | NE | 2500~3200 |
|  | *Aleuritopteris leptolepis* | LC | 2500~4500 |
|  | *Aleuritopteris subvillosa var. tibetica* | LC | 4300 |
|  | [*Onychium cryptogrammoides*](https://www.plantplus.cn/info/Onychium%20cryptogrammoides) | LC | 2500~3200 |
|  | *Onychium japonicum var. lucidum* | LC | 2300 |
|  | *Onychium tibeticum* | LC | 2300 |
|  | [*Cheilanthes nitidula*](https://www.plantplus.cn/info/Cheilanthes%20nitidula) | LC | 2900 |
|  | *Cheilanthes tibetica* | NE | 3600~4600 |
|  | [*Aleuritopteris albofusca*](https://www.plantplus.cn/info/Aleuritopteris%20albofusca) | LC | 2500 |
|  | *Adiantum breviserratum* | DD | 3200 |
|  | *Adiantum capillus-veneris* | LC | 800 |
|  | *Adiantum refractum* | LC | 2000 |
|  | *Adiantum edgeworthii* | LC | 2000 |
|  | *Adiantum fimbriatum* | LC | 3100~4150 |
|  | *Adiantum myriosorum* | NT | 2900 |
|  | *Adiantum pedatum* | NT | 2900~3400 |
|  | *Adiantum roborowskii* | LC | 2000~3500 |
|  | *Adiantum tibeticum* | DD | 2800~3200 |
|  | *Adiantum venustum* | DD | 2000~2100 |
|  | *Coniogramme affinis* | LC | 2400~3500 |
|  | *Coniogramme fraxinea* | LC | 2100~2300 |
|  | *Coniogramme intermedia* | LC | 2900 |
|  | *Coniogramme intermedia var. glabra* | LC | 2900 |
|  | *Coniogramme procera* | LC | 2000~3600 |
|  | *Paragymnopteris bipinnata* | LC | 2200 |
|  | *Paragymnopteris bipinnata var. auriculata* | LC | 2300~2400 |
|  | *Paragymnopteris delavayi* | LC | 2700~4600 |
|  | *Paragymnopteris marantae* | LC | 2400~4100 |
|  | *Paragymnopteris sargentii* | LC | 2600 |
|  | [*Paraceterach vestita*](https://www.plantplus.cn/info/Paraceterach%20vestita) | LC | 2800~3900 |
|  | [*Haplopteris flexuosa*](https://www.plantplus.cn/info/Haplopteris%20flexuosa) | NE | 2500~2750 |
|  | *Haplopteris doniana* | LC | 2100~2300 |
|  | *Haplopteris himalayensis* | DD | 2300~3400 |
|  | *Haplopteris linearifolia* | DD | 2300~2500 |
|  | *Haplopteris mediosora* | DD | 2600~2900 |
|  | *Vittaria zosterifolia* | NE | 800 |
| Selaginellaceae | *Selaginella albocincta* | LC | 3800 |
|  | *Selaginella chrysocaulos* | LC | 2100~2900 |
|  | *Selaginella involvens* | LC | 2000~2900 |
|  | *Selaginella monospora* | LC | 800 |
|  | *Selaginella monospora* | LC | 800 |
|  | *Selaginella nipponica* | LC | 2400 |
|  | *Selaginella nummularifolia* | DD | 3500 |
|  | *Selaginella picta* | LC | 850 |
|  | *Selaginella pulvinata* | NT | 2600~4200 |
|  | *Selaginella sanguinolenta form. kantzensis* | NE | 2100~2500 |
|  | *Selaginella tibetica* | DD | 2400 |
|  | *Selaginella vardei* | LC | 2200 |
|  | *Selaginella vaginata* | LC | 2000~3700 |
| Thelypteridaceae | *Cyclogramma tibetica* | DD | 1530 |
|  | *Cyclosorus aridus* | LC | 1200 |
|  | *Cyclosorus medogensis* | NE | 1000 |
|  | *Cyclosorus papilio* | LC | 800 |
|  | *Cyclosorus truncatus* | LC | 800 |
|  | [*Glaphyropteridopsis erubescens*](https://www.plantplus.cn/info/Glaphyropteridopsis%20erubescens) | LC | 2000~2300 |
|  | *Leptogramma himalaica* | DD | 2500 |
|  | *Macrothelypteris ornata* | DD | 850~1000 |
|  | *Metathelypteris uraiensis var. tibetica* | DD | 1700 |
|  | *Phegopteris tibetica* | LC | 3600 |
|  | *Pronephrium lakhimpurense* | LC | 650 |
|  | *Pronephrium nudatum* | LC | 1000~1200 |
|  | *Pseudocyclosorus canus* | DD | 900~1800 |
|  | *Pseudocyclosorus pectinatus* | NE | 1400~2300 |
|  | *Pseudocyclosorus tylodes* | LC | 1000 |
|  | *Pseudocyclosorus zayuensis* | DD | 2010~2100 |
|  | *Pseudophegopteris brevipes* | DD | 2330 |
|  | *Pseudophegopteris levingei* | LC | 2300~2960 |
|  | *Pseudophegopteris microstegia* | LC | 2300~2400 |
|  | *Pseudophegopteris pyrrhorachis var. glabrata* | LC | 2500~3000 |
|  | *Pseudophegopteris tibetana* | DD | 2010 |
|  | *Pseudophegopteris yigongensis* | DD | 2500 |
|  | *Pseudophegopteris zayuensis* | DD | 2100 |
| Woodsiaceae | [*Physematium elongatum*](https://www.plantplus.cn/info/Physematium%20elongatum) | DD | 3400 |
|  | *Woodsia alpina* | DD | 3900 |
|  | *Woodsia lanosa* | LC | 3500~4500 |
|  | *Woodsia rosthorniana* | LC | 3000 |

**Supplementary Table 2.** Summary of correlation among climatic variables, species richness and elevation. The colored showed the significance values.

| **Climatic variables** | | | | | | | | | |
| --- | --- | --- | --- | --- | --- | --- | --- | --- | --- |
|  | **GP** | **GDD5** | **GDD0** | **MI** | **DI** | **MAP** | **SS%** | **MAT** | **S** |
| **Elevation** | -0.98 | -0.98 | -0.99 | -0.41 | 0.12 | -0.81 | 0.86 | -1.00 | -0.02 |
| **GP** |  | 0.94 | 0.97 | 0.46 | -0.18 | 0.84 | -0.86 | 0.99 | 0.12 |
| **GDD5** |  |  | 1.00 | 0.23 | 0.07 | 0.68 | -0.74 | 0.97 | -0.16 |
| **GDD0** |  |  |  | 0.31 | -0.02 | 0.75 | -0.80 | 0.99 | -0.07 |
| **MI** |  |  |  |  | -0.95 | 0.85 | -0.78 | 0.41 | 0.74 |
| **DI** |  |  |  |  |  | -0.66 | 0.56 | -0.12 | -0.77 |
| **MAP** |  |  |  |  |  |  | -0.98 | 0.81 | 0.50 |
| **SS %** |  |  |  |  |  |  |  | -0.85 | -0.43 |
| **MAT** |  |  |  |  |  |  |  |  | 0.03 |
